# Maternal and Child Survival in Haor Region in Bangladesh. An Analysis of Fathers’ Capabilities to Save the Future

**DOI:** 10.3390/ijerph17165781

**Published:** 2020-08-10

**Authors:** Barnali Chakraborty, Shrinivas Darak, Hinke Haisma

**Affiliations:** 1 BRAC James P Grant School of Public Health, BRAC University, 68 Shahid Tajuddin Ahmed Sharani, Mohakhali, Dhaka 1212, Bangladesh; 2 Population Research Centre, Faculty of Spatial Sciences, University of Groningen, Landleven 1, 9747AD Groningen, The Netherlands; h.h.haisma@rug.nl; 3 Prayas Health Group, Pune, Maharashtra 411 004, India; shirish@prayaspune.org

**Keywords:** capability approach, women’s health, child growth, gender, health communication, health care seeking

## Abstract

Maternal and child survival is a major public health problem in haor areas in Bangladesh. Fathers feel responsible as expressed by their capability “to save the future”. Using the Capability Framework for Child Growth, we aimed to identify what contextual factors underlie a father’s real opportunities to secure a safe delivery, including social norms and beliefs. Parents from households having children less than two years old were asked to participate in two rounds of qualitative research. In total, 25 focus group discussions and eight in-depth interviews were conducted. Late admission to health facilities emerged as the overarching disabling factor for fathers’ capability to save the lives of mothers and children. Poor communication about the mother’s health condition between spouses and fear for caesarean birth were underlying this late admission. In addition, inadequate advice by local doctors, underdeveloped infrastructure, and seasonal extremities contributed to late admission to health care facilities. The participants indicated that mother’s autonomy in haor to seek health care is a constraint. This capability analysis points towards relevant interventions. In addition to the need for an improved health infrastructure, programs to improve maternal and child survival in haor could focus on the gendered household responsibilities and poor communication between spouses.

## 1. Introduction

Despite commendable progress in maternal and children under-five survival over the last decades [1,2], mortality rates remain high. According to recent estimates by the United Nations (UN) Inter-Agency Group for child mortality estimation, the under-five child mortality rate in Bangladesh was 30 per 1000 live births in 2018, a 66% decrease since 2004 [1,2]; but inequality across regions is persistent. In contrast, maternal mortality rates remain stagnant at 196 per 100,000 live births since 2010 to 2016. Around two-thirds of under-five mortality are due to neonatal and infant mortalities as a result of inadequate access to quality health care during pregnancy, childbirth or the postnatal period [1,3,4,5]. The main causes of maternal mortality include obstetric complications such as eclampsia, hemorrhage, prolonged labor or abortion [3,6]. Although biomedical and socio-epidemiological frameworks, such as the analytical framework for child survival by Mosley and Chen [7] and the three-delay model by Thaddeus and Maine [8], have helped us elucidate the determinants of maternal and child mortality, a deeper understanding of the root causes is needed to further reduce the persistently high numbers in many countries, including Bangladesh. We used the capability approach as originally developed by Amartya Sen and Martha Nussbaum [9,10,11], and further developed by us for child growth [12,13] to identify such root causes.

The burden of maternal and child mortality is particularly high in rural areas [1,6,14]. In Bangladesh, one of the most remote regions constitutes the haor region located in the north-eastern part of the country, spreading over Sunamganj, Sylhet, Habiganj, Moulavibazar, Kishorgonj, Netrokona, and Brahminbaria districts. The areas are geographically vulnerable because of long-term floods during the wet season (roughly for six months) and post flooding effects during the dry season. Over 19 million people reside in this region and more than half of the population are poor, marginal farmers [15,16]. Around 95% of women deliver at home with the assistance of unskilled birth attendants [17]. Although there are no statistics available for the haor region specifically, maternal and child mortality is likely to be very high.

Involvement of men as the husband or expectant father throughout the period of pregnancy and delivery is crucial in ensuring access to improved maternal health care services and in achieving improved maternal health outcomes [18,19,20,21]. Their emotional and materialistic supports, such as arranging transportation, getting financial resources, and sharing information during pregnancy and childbirth, are instrumental in increasing access to quality health care services during pregnancy, childbirth, and the postnatal period [19,21,22,23]. However, to be able to play such roles efficiently, they need to have adequate access to the required resources, a supportive societal environment, knowledge, and adequate love for their wife [19,23]. In haor areas, lack of resources, extreme weather conditions, and parents’ social norms underlie the high mortality rates both for mothers and their children. Fathers of haor describe their role in achieving a healthy delivery as the capability “to save the future” and identified it as a key dimension of child growth [24]. In this paper, we focus on this particular paternal capability “to save the future”, using the Capability Framework for Child Growth (CFCG) for its further analysis. We go beyond a focus on resources and health outcomes and intend to identify what contextual factors underlie a father’s real opportunities to secure a save delivery, including social norms and beliefs.

## 2. Materials and Methods

### 2.1. Conceptual Model

This paper is embedded into larger research on multi-dimensional child growth [12,13,25] using the Capability Framework for Child Growth as a conceptual model. The concept of multi-dimensional child growth is modeled based on Sen and Nussbaum’s work on the capability approach [9,11,26]. Instead of focusing on utilities or resources needed to ensure health outcomes, the capability approach asks what a person is able to do or to be in his or her societal and environmental context in order to lead a life that s/he values [9,26]. It is, therefore, a normative framework that takes into account context and values. According to this framework, children draw their entitlements from the microsystem where caregivers and family members play important roles in shaping children’s capabilities to grow well. That is, children’s capabilities are dependent on parents’ or caregivers’ capabilities. A parent’s capability is shaped by the availability of resources (for example, money and health care services) and by the conversion factors (at the personal, societal or environmental level) that facilitate or restrict the translation of the resources into effective capabilities. The framework finally makes explicit the role of agency that is defined as the ability to pursue one’s goal. It leads one to transform the capabilities into the valuable outcome using the available resources. The other layers, such as meso, exo, and macro system, highlight the linkages across two or more settings influencing a child’s development indirectly such as doctors, teachers, belief systems, social norms, culture, and institutional settings. Initially, and as published elsewhere, we identified capabilities for healthy child growth at the child, parental, and household level. A father’s capability to save the lives of mothers and children, expressed as “the capability to save the future” emerged as an important opportunity to secure maternal and child survival [24]. Maternal and child survival are thus some of the core capabilities for healthy child growth.

### 2.2. Study Area, Population, and NGO Involvement

**Figure 1:**
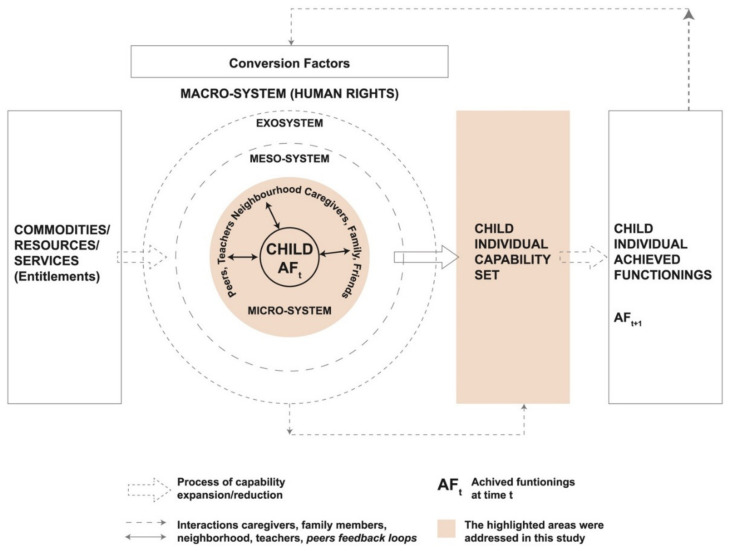
Capability Framework to Child Growth by Yousefzadeh et al. [12]. Reprinted by permission from Springer Nature: Springer Nature, Child Indicators Research, Copyright (2018).

Three sub districts, Derai, Baniachang and Ashtagram, were selected purposively for this study respectively from Sunamganj, Habiganj, and Kishorganj districts. A range of haors of varying depths are spread over these areas that are heavily flooded during the wet season. The populations of Derai, Baniachang, and Austagram are around 244,000, 300,000, and 152,000, respectively [27]. The people living in these areas attempt to protect their houses from flooding by building them slightly above the ground in clusters known as ‘hati’ [24]. Each hati comprises 20 to 50 houses. BRAC, a non-governmental organization (NGO), has implemented integrated development programs in Derai and Baniachang to provide need-based interventions to poor communities since 2013. These interventions have a multi-sectoral approach and include measures to improve agricultural productivity, food security, access to healthcare, and nutrition security. The program offers services on microfinance, boat school facilities for children, construction of tube wells and toilets, light counseling on health, and nutrition and paramedical services in some selected areas. The program also includes the option for boat delivery facilities in some areas, but these services are yet to be implemented. Ashtagram is geographically harder to reach than Derai and Baniachang where activities of BRAC or other NGOs are limited. A small-scale BRAC microfinance program operates there to provide loans to the communities within their reach. Thus, these programs and interventions are utility-based, referring to commodities, resources, and services (first column in Figure 1).

### 2.3. Study Design and Participants’ Recruitment

The study was conducted using a participatory qualitative research design. Parents from households having children less than 2 years old were asked to participate in two rounds of research, as this age group is vulnerable in achieving their growth potentials. In both rounds, the fieldwork was conducted during the dry season as during the wet season men mostly migrate to other areas for earning their livelihood. The field staff of BRAC, local representatives of the communities working for the Government of Health, Family and Welfare services, villagers, and the participants assisted the research team in finding the locations and the households from which the participants were selected. In the first round (from December 2016 and January 2017), eight focus group discussions (FGDs) were facilitated with the parents (four with mothers and four with fathers) in different villages of Derai and Baniachang to identify the capabilities underlying multi-dimensional child growth in haor areas. Around six to ten participants participated in each of the FGDs. In-depth interviews (IDIs) were conducted with four fathers and four mothers to complement the information at the individual level. The IDIs were conducted at the participants’ homes and the FGDs were facilitated in the household yard, a school classroom or at the participants’ homes that provided sufficient privacy. Fathers did not attend the interviews with mothers and vice versa. The fathers and mothers were selected from different households to get diversity in the collected information. The discussions and interviews were then transcribed and analyzed, and findings were used for the preparation of the second round.

The second round of the study was done in the month of February 2019 in Derai and Ashtagram to validate the findings of the first round. In this round, 17 focus group discussions were facilitated (10 FGDs with mothers and 7 FGDs with the fathers). The number of participants in each of the FGDs was similar to that of the first round. To facilitate discussions, we selected household yards or participants’ homes as we did in the first round. The process of data collection continued until data saturation was achieved (i.e., until the moment that the responses of the participants did not result in any new codes or themes) [28].

### 2.4. Data Collection, Coding, and Analysis

An interview guide with open-ended questions was used to facilitate FGDs and the IDIs in the first round. For FGDs the questions were structured to explore the context of healthy child growth from a communities’ perspective and get information on community opinions. For IDIs the questions were designed to explore personal experiences and perceptions and get people’s individual stories. The interview guides were prepared using the CFCG as a conceptual model and were further informed by literature review on child health, growth and wellbeing [12,29,30] and by discussions with researchers and experts in these areas. The interview guides were pilot tested in the study areas with the aim to get information on (1) the feasibility to gather participants (particularly, the fathers) for an FGD or IDI; (2) the adequacy of the questions in terms of understanding by the communities and in collecting the required research information; (3) the length of the discussions/interviews. We facilitated four FGDs with the parents of children less than 2 years of age (2 with mothers and 2 with fathers) and conducted five IDIs (3 with mothers and two with fathers) in different haor areas of Derai and Baniachang during the wet season. The interview notes were not analysed as the main objective of the pilot testing was to develop efficient field work plans and adapt the questions relevant to the context. For example, after the pilot testing, we decided to conduct our field work during the dry season considering the feasibility of gathering the participants for FGDs. We also revised some of the questions to make them understandable to the communities. For example, initially we shaped the specific question of parental capability as ‘who is defined as a good father or a good mother’ that we later revised as ‘what kind of abilities does a father, or a mother need to provide good care to the child’. The refined interview guides used during the first round of data collection were provided with the other paper by Chakraborty et al 2020 (24).

Of particular relevance to this paper were the questions: what happens in haor when the mother or the child needs health care during pregnancy and delivery? How is this influenced by the seasonal floods, the transition phases, and post flood situations? Who usually makes the key decisions in this process? The responses of the participants were transcribed and analyzed to shape the FGD guides for the second round. The analysis was done through an iterative process of inductive and deductive reasoning [28]. The transcribed notes were processed into the N-Vivo Software (QSR International Pty Ltd., Melbourne, Australia). The coding followed two steps: for the first step, coding was done inductively, staying close to the text and identifying anything that was found relevant. For the second step, the inductive codes were matched deductively with the constitutive elements of the CFCG. We developed a single comprehensive code book for the FGDs and IDIs (as the domains explored were similar for FGDs and IDIs). The process of analyzing the data from the first round is discussed in more detail elsewhere [24].

**Figure 2:**
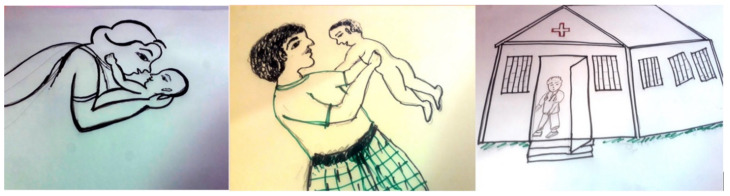
Examples of drawings demonstrated to facilitate participatory research (drawn by Debashish Chakravorty). The first two drawings on mother–child love and father–child love were generated following the clip art from the internet [31,32]. The third drawing on health care facility was generated based on the contextual experience from the first round of data collection.

In the second round, an FGD guide with drawings and pictures (see Supplementary Materials) was used to further explore the findings that emerged from the first round with the communities. For the specific research question of this paper, the participants were shown a drawing of a hospital with a doctor and asked, how do they reach the hospital when the mothers are in childbirth? (Figure 2). They were asked what kind of challenges do they face to reach the hospital in different seasonal phases and how do they address those challenges? How could their challenges be addressed and who can contribute in overcoming these challenges? We also probed their decision-making process based on their responses as needed. The focus group discussions in both rounds were moderated by the principal researcher with the support of an assistant who helped in taking notes on the discussions. The in-depth interviews were conducted by the principal researcher in a one-on-one setting to explore the individual’s experiences. Some examples of the drawings demonstrated during the participatory discussion are given below (Figure 2). Some of the drawings were generated using clip art from the Internet (e.g., mother–child love, father–child love) and some (e.g., drawing of the health care facility) were drawn based on the meaning of the capabilities that emerged from the first round of data collection. In Figure 2 the first drawing shows that a mother is expressing love to her baby. This was used to explain a mother’s capability to provide love and care to her child. The second one demonstrates a father’s love for his child and was used to discuss father’s love and care for a child. The third drawing shows the entrance of a health care facility that was used to explore the contextual factors experienced by the communities in reaching a health care facility.

Discussions from the second round were tape recorded and transcribed verbatim by the research assistants. The transcribed notes were then processed into the N-Vivo software package (QSR International Pty Ltd., Melbourne, Australia). The data were coded following the specific capability of “saving the future” identified in the first round. Inductive codes were identified and subsequently matched with the constitutive elements of the capability approach (i.e., resources, conversion factors, and agency) deductively. Responses of the participants were reviewed to see if the stories confirmed previous findings from the first round of data and if any important aspects remained unexplained in the first round. Thus, the findings of both rounds were synthesized to get a complete picture of a father’s capability to save the life of mothers and children in haor.

### 2.5. Ethical Issues

The study was approved by the ethical committee of BRAC James P Grant School of Public Health, BRAC University, Bangladesh and the research ethical committee of the Faculty of Spatial Sciences of Groningen University, the Netherlands. The participants gave written informed consent before the start of the interview and FGDs. As most of them were illiterate and were not able to read, the consent form was read to participants. In some cases, a literate participant or local person justified the consent form for everybody present and checked whether the facilitator’s reading was consistent with the writing in the consent form. Most of the participants were able to write their first name to sign the form. A very small number of participants gave their initial to sign the form as they were not able to write their name. To maintain the confidentiality of the information and identity of the participants, all data were anonymized. It was made clear to the participants that they are flexible to refuse to answer or withdraw participation at any point if they felt uncomfortable. The study participants were provided with local snacks during the interviews, and soaps as gesture of thanks for their participation.

## 3. Results

### 3.1. Participants in the Study

In total, 69 fathers participated in FGDs (23 in the first round and 46 in the second round) and four participated in IDIs (first round). The age of the fathers ranged from 21 to 40 years. Most of the fathers stated they mainly do paddy cultivation when the fields are not flooded, while others mentioned working in small business, such as selling betel-nuts, vegetables, or working as day laborers (rowing boat, carpenter, barber, earth digging, mason, and rickshaw puller). When the areas are flooded, they move to other areas and work as daily laborers. Those who stay in haor usually earn by fishing or grocery business, otherwise they stay jobless. The majority of the fathers received no or little formal education. Some fathers had gone to primary school but did not complete it; others completed primary school, and a few went to high school or completed Arabic (Islamic) education at the primary level. The majority of fathers were Muslim, with Hindu being a minority.

In addition, 113 women participated in FGDs (33 in the first round and 80 in the second round) and four women participated in IDIs (first round). The age of the mothers ranged from 16 to 35 years. The mother’s main task was to do household chores and take care of the children. About one-third of the participants never went to school and were able to sign only (i.e., write their name). Some completed primary education, while others initiated primary education but did not complete it. A few went to high school and a few mentioned receiving Arabic (Islamic) education at the primary level. The majority of mothers were Muslim, with Hindu being a minority.

### 3.2. A Capability Analysis of “Saving the Future” in Haor

In this section, we will start with a description of fathers’ capabilities to save the lives of mothers and children as identified in an earlier stage of this research project [24]. This will be followed by an in-depth analysis of this capability using the constitutive elements of the CFCG (Figure 1). As mentioned earlier, the CFCG was used to understand how an individual’s capability is shaped by the availability of resources and facilitated or constrained by conversion factors. In this section, we intend to understand how a father’s capability to save the lives of the mothers and children is deprived in the haor context, and what would be needed for its improvement.

#### 3.2.1. Being Able to Save the Lives of Mothers

Fathers indicated that they are tense when there is a pregnant woman in the family; they worry whether she will survive or not as there is no equipped health facility for seeking prenatal or delivery care in their area. They referred to this precarious situation around delivery as ‘kaal bipod’ meaning ‘a danger of death’. They also referred to an equipped and skilled health facility as ‘a shelter’ that they miss during this danger period. A father described this situation in the following way:

*“This is our main problem, particularly when there are women (pregnant women), you know! Danger comes once, it is called kaal bipod. During this danger time, we don’t get any shelter, we fall in huge trouble.”*

FGD, Fathers, Perua, Derai, 2nd round.

#### 3.2.2. Being Able to Save the Lives of Children

The participants stated that many pregnant women in haor had experienced multiple fetal, neonatal, and infant deaths because of delivery complications or postnatal health complications such as pneumonia, fever, or breathing problems. Fathers’ worries in this regard are clearly expressed in the following quote [24]:

*“See, before marriage people dream of having a beautiful wife, then after getting married people dream of having babies. (Someone starts laughing) … No do not laugh! Why do you laugh? When we talk about the important aspects of marriage you laugh! See these children are our future. Now to get this future, if the future along with the person through whom the future is delivered, die, it takes us through the saddest experience of life. The saddest thing more than anything!”*

FGD, Fathers, Golbogi, Baniachang, 1st round

#### 3.2.3. Endowments Underlying the Capability “to Save the Future”

Further analysis of the capability “to save the future” using the constitutive elements of the CFCG (Figure 1) revealed the lack of certain commodities and services as important causes underlying the deprivation of this capability. For example, a nearby hospital or equipped health facility, money, transportation, and skilled health care services were mentioned as important resources; lack of these services resulted in the deprivation of a father’s capability to “save the future”. As the added value of the CFCG in relation to other frameworks is mostly at the level of conversion factors and agency, we will not discuss the emergence of these entitlements in much detail here. We briefly elucidate on them to provide context to the subsequent sections. For example, the participants said that they do not have the infrastructure for lifesaving medical facilities, such as oxygen for cesarean section, that led them to seek health care from the unskilled local health care providers and traditional birth attendants. The participants also mentioned about fathers’ limited affordability for health care costs as they lack the capability to earn, particularly when there is a flood. Therefore, they mostly observe the health conditions at home and are late in seeking quality treatment for mothers or children and therefore fail to save their lives. They also highlighted the importance of having sufficient suitable vehicles to take the patient to the hospital quickly during emergency situations. In haor a manual vehicle is used such as the thelagari or hammock. Thus, many mothers and children die on the way as it takes too long to reach the hospital located at the district level. They further raised their concern about the skills of the local doctors and birth attendants who fail to identify or treat the health problem, again putting the mother or child at risk of dying. More information about the entitlements in relation to multi-dimensional child growth has been described elsewhere [24].

### 3.3. Conversion Factors: Barriers and Facilitators of Father’s Capability “to Save the Future”

Conversion factors that emerged from the IDIs and FGDs underlying the deprivation of a father’s capability to save the future relate to gender issues, social norms and cultural beliefs, seasonality, and infrastructure. Key conversion factors include lack of communication about a mother’s health between the spouses, inadequate advice by local doctors, fear of caesarean delivery, and reliance on God.

#### 3.3.1. Lack of Communication About a Mother’s Health

The participants mentioned that a father’s capability to save the lives of mothers and children is deprived due to poor communication between the spouses. Mothers do not always express their pregnancy complications to their husband or other family members at an early stage. This results in late admission of their pregnant wives to the health facility and they thus fail to save the lives of both mothers and the fetus or neonates. When the condition deteriorates and becomes visible to all, then attempts are made for the affected mothers to get admitted to the hospital at the last minute. A story of a pregnant mother as stated by the participants illustrates this information gap:

*“…She had an infection on one side of the bottom of her belly but she did not tell anyone about it. She didn’t tell anyone! Four months passed and when she was in her tenth month of pregnancy, the condition deteriorated and she was totally infected. …they admitted her to the government hospital at Banglabazar. The doctor didn’t give her any medicine. He said: ‘it is delivery pain; both baby and mother could die if they would be given any medicine’. That woman’s condition got worse but the doctor didn’t want to take any risk. They returned home, and went to another doctor. They bought medicine for the fever. After having that medicine, the baby probably died. It was stuck inside the mother’s womb! … Then they took out the baby through caesarean section, but both the mother and the baby died.”*

FGD, Mothers, Basakarach, Derai, 1st round

#### 3.3.2. Inadequate Advice by Local Doctors

The participants mentioned that another reason for late action by the fathers in case of critical conditions during pregnancy lies in irrational advice given by local doctors. For example, when a mother gets edema and develops convulsions with frothing (a form of eclampsia), the husbands are advised by the local doctors not to give medicine as it may affect the life of the fetus. A mother stated her sister’s story in the following way:

*“She got water throughout the body. The body was swelled by water. The husband didn’t treat her. He said if I treat her for the body water the baby will die. Because the doctor said not to treat her for this, it may kill the baby. That’s why he didn’t do it. Then she had caesarean delivery, after 3 days my sister died.”*

FGD, Mothers, Sarail, Baniachang, 1st round

#### 3.3.3. Fear of Having a Hospitalized Delivery

The participants said that in the context of haor the social norm is to have a home delivery, as institutional delivery mostly implies having a caesarean section. They (both mothers and fathers) pointed out they were scared of having a caesarean delivery, and concerned about the quality of care, painful stiches on the belly, extra costs, and post-caesarean effects. They perceive that having a caesarean delivery will make them unable to perform their household responsibilities, such as childcare, cooking, collecting water, preparing cow dung for fuels and for coating the floor, as well as participating in post-harvest activities such as boiling and drying of harvested paddy. This reflects the gendered division of household tasks. A participant stated this in the following way:

*“We first try at home, all observe, the patient herself observes. In our area home delivery is better. For example, here the women need to do heavy work. If they go for caesarean they won’t be able to do such heavy works. We all want to have normal delivery at home.”*

FGD, Mothers, Chandipur, Baniachang, 1st Round

#### 3.3.4. Reliance on God’s Mercy

The participants mentioned that some people in haor feel shy to go to hospitals to seek prenatal or delivery care as it is not common in haor areas. They rely on God’s mercy; although this was reflected mostly among the Muslim communities. Only when the condition deteriorates, do they decide to go to the hospital. Therefore, they reach the doctor too late and the doctors fail to adequately treat the critical condition. A father shared what he values in the following way:

*“I didn’t take her for any checkup. One advised me to do ultrasonography, but I didn’t do because I have the confidence. Good or bad will be justified by God, what’s happening in our mind all are known by God”*

IDI, Father, Talibpur, Baniachang, 1st round

#### 3.3.5. Poor Transportation Due to Flooding or Receding Water

The participants said that reaching a health facility on time is always a concern in haor because of the underdeveloped infrastructure and seasonal extremities. When haor areas are flooded, participants indicated they would take the patient to the health facility by boat. However, if the wave of the flood rises high with strong wind, they need to wait till the weather calms down. A father stated that he had a stillbirth baby as he could not take the mother to the hospital due to stormy weather during her delivery pains. His statement is quoted below:

*“It was during the wet season, there was a high wave with storm, during that storm the baby’s mother had delivery pain and I was not getting a boat. There were huge waves in the river, I could not manage any boat. Thus, a day went by; in the end, I couldn’t reach the hospital.”*

FGD, Fathers, Rafinagar, Derai, 2nd round

The distance between roads and houses is another problem mentioned by participants in relation to the underdeveloped infrastructure of haor. They said in haor areas the roads are far from their house; most are on the other side of the river. In addition, the local vehicles such as Honda/tempo that are available for getting from the village/hati to the road are not suitable to carry a patient. Therefore, they need to carry the patient manually a certain distance to reach the road and, in some areas, they need to cross the river to reach the road to get a vehicle. A mother described the situation in the following way:

*“The vehicle is not available nearby the door; in other areas, the vehicles are available nearby the door. The reason is, there is no road. First a boat is required, then a vehicle is hired… You need to take this route (to reach the road where the vehicle becomes available) at least. You see, even if it is dry, there is no tempo (local motor vehicle with three wheels) available. A honda is available, but you can’t take her with a honda. Then she is to be taken by placing her on a hammock.”*

FGD, Mothers, Dalarkandi, Ashtagram, 2nd round

They also raised their concern about post flood effects. They said when the flood water starts to recede or enter, the roads turn muddy and the vehicles stop moving through the muddy tracks. When it gets completely dry, most of the areas end up with broken roads. They said in such conditions their mobility is also affected and they carry the patient using manually-pulled local vehicles or a hammock. Some stay at home without going to a hospital to avoid the bumping of the broken roads. A father described this situation in the following way:

*“…Now if half of us stay inside our homes we will remain well but if we move through the street, you know, … it is very difficult. The bumping of the vehicle makes the condition serious). If your disease is 50%, it will increase by 25% more. The condition gets worse…”*

FGD, Fathers, Golbogi, Baniachang, 1st round

The participants mentioned that sometimes a patient in a critical condition is taken to several referral facilities from the sub-district to district level. As the infrastructure is not good, their travel includes changing several vehicles: partially they travel on foot, partially by boat, partially by rickshaw or by another motor vehicles. At the end, they are late to reach the appropriate facility and either the mother or child or both die before receiving treatment. A mother shared the story of her neighbor/relative in the following way:

*“The baby was okay after the delivery. At the fifth day, it had pneumonia. After searching rigorously, they got a boat and took the baby. Next, they got a van, then they were required to move to a motor vehicle, but they didn’t get it. Then finally they got the vehicle but they were late. They got it around at 1–2 pm. Then they took the baby to the hospital. There the baby didn’t get the treatment. The patient was seriously ill. Now at the last minute, they took the patient to Habiganj. From there they had to take the baby to the doctor’s house but in the meantime the baby died, finally they couldn’t reach the doctor with their baby.”*

FGD, Mothers, Sarail, Baniachang, 1st round

Thus, the above analysis of the participants’ quote indicates how various conversion factors disable a father’s capability to save the lives of mothers and children. Although different in their nature (i.e., social norms, cultural beliefs, or seasonal effects on transportation possibilities), they all relate to timely and adequate admission to a health facility.

### 3.4. Gender Issues and Mothers’ Autonomy

The participants indicated that maternal and child survival is a collective goal for haor communities. However, to achieve this goal, fathers play the role of an agent on behalf of the mothers as the mothers in haor are neither financially independent nor are the societal arrangements in favor of them. Their gender-specific roles keep them confined to indoor activities with limited mobility to the outside world. They do not have the knowledge on how to reach a health facility located far from their village. They just act as an informant about their health condition—and this is often compromised as indicated in the above section on poor communication between the spouses—based on which the husband decides what to do or how to address the problem. A father describes this in the following way:

*“Suppose we know how to go to another village, how to go to Mordanpur, Muradpur, but not all know how to reach there, particularly the women don’t know how to reach the place. There is one (health care centre) in Auladpur, and the other in Mordanpur. We need to walk to bring the doctors from there”*

FGD, Fathers, Makhonia, Baniachang, 1st round

The participants indicated the importance of women’s autonomy in handling the situation. They said that if the hospitals were built at the village level and if the number of boats and suitable vehicles with drivers were made available within their area, then the women would not have to wait for men’s support before admitting a patient to the hospital. In that way women’s autonomy would be improved and they would be able to reach health care facilities by themselves or could help each other in handling the situation. A mother expressed her view regarding the presence of vehicles and how this enhances women’s autonomy and it contributes to how women could assist each other in their access to health care in the following way:

*“…to carry someone by hammock requires four people. Four people are not always available. If there would be a vehicle and a driver, the women could take the patient (to the hospital)”*

FGD, Mothers, Dalarkandi, Ashtagram, 2nd round

The participants also suggested building embankments and constructing roads and bridges at higher altitudes to protect the areas from floods or for creating earning opportunities in haor areas. They said that if the women were provided training on preparing handloom materials, rearing poultry and livestock and the men were given training on agriculture, conditional financial support at repayable interest, they would be able to improve their affordability. The participants perceived that if both men and women could earn then they would be able to share the financial responsibilities. That way, the women would be able to cover their health care costs by themselves. A father from Derai emphasized the importance of a mother earning in the following way:

*“…if the mother could do other work, could earn, then she would be able to spend it for health care, she could follow a doctor’s instructions, thus she could keep herself healthy”*

FGD, Fathers, Dhalbazar, Derai, 2nd round

As discussed before, gender norms underlie the fear to be hospitalized, as a caesarean will take time to recover and cause a mother to not be able to take up her household chores soon after giving birth.

Thus, the above statements indicate how gender issues play a role in several of the conversion factors mentioned earlier. Addressing these issues could contribute to improve women’s autonomy and help to reduce maternal and child survival collectively both by men and women.

## 4. Discussion

**Figure 3:**
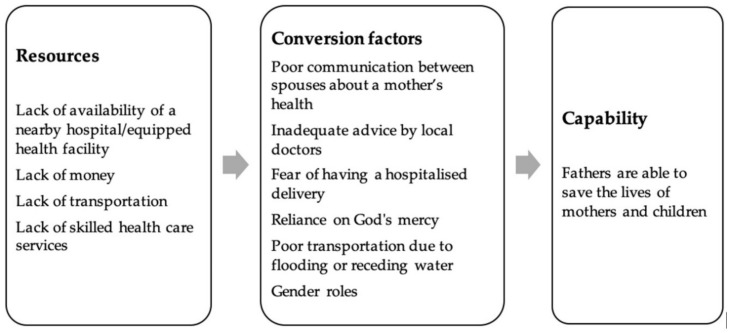
Summary of the findings in light of Capability Framework for Child Growth (CFCG).

In this paper, we analyzed a father’s capability to save the lives of mothers and children in the light of the CFCG [12]. Although we focused on this one dimension of child growth [24], this capability is interrelated to a father’s other capabilities, for example, the capability to earn and the capability to be mobile at different seasonal phases to admit the patient to the hospital. Deprivation in one of those capabilities affects the capability to save the lives of the mothers and the children. This interlinkage between capabilities has also been described by other capability scholars [29,30,33]. The analysis of the capability to save the lives of mothers and children using the CFCG has provided us with a rich understanding of what underlies the poor achievement of maternal and child survival in the context of haor in Bangladesh. While other frameworks, such as the child survival model [7] or the three-delay model [8] have been instrumental in understanding the determinants of maternal and child survival, the CFCG provides a deep understanding of what underlies these determinants. The conversion factors that compromise a father’s capability to save the lives of mothers and children all pointed towards a delay in admission and/or attendance; but the added value of using the CFCG for its further analysis is that we learned that this is about gender roles and poor communication between spouses, irrational advice by local doctors due to cultural beliefs, fear of hospitalized delivery, reliance on God’s mercy, and infrastructure problems as a result of flooding or receding water. The findings also indicate that women in haor lack agency and are dependent on their husband’s actions in saving their lives. Thus, more than conventional biomedical frameworks, the application of the capability approach allowed a rich analysis of the responses of the participants from a multidimensional angle and included external factors that affect how resources can or cannot be transformed into people’s real opportunities. Our analysis of maternal and child survival in this study is unique both from the use of the CFCG as a conceptual model, as well as its emic perspective (how local people perceive and interpret). The findings are summarized below (Figure 3).

Poor infrastructure and lack of nearby health facilities emerged as another disabling factor for fathers’ capability to save the lives of mothers and children. In Bangladesh, 3% of the gross domestic product (GDP) is spent on health care; in remote areas such as haor, health facilities are geographically unevenly distributed and there is an inadequate number of qualified doctors. The overall figure for Bangladesh in terms of medical staff is four physicians per 10,000 people and two nurses per 10,000 people, most of which are concentrated in urban secondary and tertiary hospitals [34]. Furthermore, in Bangladesh, around two-thirds of the total health expenditure come from out of pocket payments [5,35,36]. Therefore, people with little access to financial resources tend to avoid going to the health care facility until an emergency arises as we see in the haor context. So rationally, people residing in hard-to-reach areas like haor are less likely to have access to skilled health care services. Moreover, beyond a deprivation in available resources (e.g., money, doctors, and transportation), socio-cultural or contextual aspects have significant implications for the preparedness for emergency situations in the process of seeking health care for childbirth [37,38,39]. For example, in haor, the village doctors discourage medication during pregnancy even if the condition would be critical because of their local belief system. Similarly, seasonal extremities, such as strong wind and high waves during floods restrict the use of boats in seeking health care from tertiary facilities. Thus, the application of the CFCG helped us to uncover the societal, cultural, and environmental barriers that facilitated or restricted the father’s capability to save the lives of mothers and children in haor communities. This calls for interventions that are specifically targeted to the underlying causes of maternal and child mortality in the haor context.

The findings help to understand how we can fill our knowledge gaps by giving voice to local communities. They point towards new strategies to further improve maternal and child survival in the particularly vulnerable context of haor. For example, the poor communication between spouses as an underlying factor for maternal and child mortality calls for action in which both spouses should be equally and possibly simultaneously targeted. Fear for hospitalized, caesarean births seems to be part of the gendered society in haor where women need to be productive as soon as possible after birth and resume their household responsibilities. This is the typical scenario of any patriarchal society that imposes unequal distribution of responsibilities on both men and women. While maternal and child survival is a joint responsibility of both fathers and mothers, the lack of agency of women currently makes this the sole responsibility for the father. The mothers have no voice in negotiating their role, but at the expense of their socially expected gender roles.

Gender issues, as particularly evident from women’s poor autonomy in health care seeking or opting for hospitalized birth, explain why the capability to save the lives of mothers and children was mostly a father’s responsibility. Having employment and control over their financial resources could increase women’s autonomy and the likelihood of receiving skilled health care during pregnancy and delivery [20,40,41,42,43]. In haor, women’s autonomy is affected by their lack of access to financial resources, availability of women-friendly social arrangements for health care after caesarean delivery, limited knowledge on how to find out the location of the health facility, and by their gendered roles restricted to indoor activities. The feedback of the communities indicated that improving women’s autonomy could positively contribute to enhance the capability of both fathers and mothers to address the problem of maternal and child survival in haor areas. It is also indicative that development, such as building hospitals in the village, constructing embankments and roads at higher altitudes, and increasing the availability of earning opportunities both for women and men would be ways to improve women’s autonomy in haor. However, to ensure the effectiveness of such developments in improving women’s autonomy in the patriarchal setting of haor, women would have to be socially and culturally supported to expand their movement beyond their traditional roles and responsibilities.

## 5. Conclusions

This paper pioneers the application of the Capability Approach to Child Growth (CFCG) focusing on fathers’ capabilities to save the lives of mothers and children in haor areas of Bangladesh. The findings of this study not only generate insights on individual and societal resources but also on societal, cultural, and environmental factors that shape this particular capability of the fathers in haor. The findings also shed light on diverse ways of improving women’s autonomy that are built on communities’ views to enhance the collective capabilities of both fathers and mothers in addressing the problem of maternal and child survival. Our findings could contribute to raising discussion among policy makers and program planners to identify a context specific approach in sensitizing the communities in realizing the shared responsibilities of both fathers and mothers in assuring maternal and child survival. Such advocacy and sensitization interventions will be effective when the resources and instruments needed for seeking health care by the women are made available to them. As gendered differences are socially constructed, it is important that the policy instruments and interventions at the community level are shaped in that direction. A cross-departmental collaboration beyond the health sector would be needed to initiate this.

## Supplementary Materials

The following are available online at https://www.mdpi.com/1660-4601/17/16/5781/s1.
