# Supplementary material for: Maternal and Child Survival in Haor Region in Bangladesh. An Analysis of Fathers’ Capabilities to Save the Future
Source: Int J Environ Res Public Health. 2020 Aug 10;17(16):5781. doi: 10.3390/ijerph17165781 (PMC7460361; doi:10.3390/ijerph17165781)
Supplement: Supplementary file 1 [file ijerph-17-05781-s001.pdf]

International Journal of Environmental Research and Public Health

Research tool: Second Round

Participatory research tools

PhD project: A capability approach to child growth in haor areas of Bangladesh

Consent Form

Introduction

Hello, my name is ………………………………………………………. I came here to conduct a participatory research with the people in haor, particularly with the parents of u-2 children, to understand how the children of these age group can grow in a healthy way in haor. The project is a joint effort of BRAC School of Public Health, BRAC University, and Groningen University of Netherlands. At the beginning of the project, we went to different haor areas to see the seasonal context in-person and discussed with the people of haor how it affects people's life and their children's health and wellbeing. That helped us to identify different issues that parent face in haor in relation to their children's growth. We realised it is now time to validate those findings with you to understand whether we have correctly captured your views and ensure that we have not missed out any urgent agenda to be addressed. This process will help us to rightly convey the messages of haor dwellers to the action planners who intend to improve their existing efforts.

The discussion may take around 60 minutes. To facilitate the discussions, we will use some pictures. We would like to take notes and record your responses/discussions if you kindly allow. Your participation is completely voluntary and you may withdraw from the study at any point without giving any reason if you feel uncomfortable. However, we value your participation in this research.

We haven't come to you in providing any service or advice or money rather we intend to work in finding out long term solutions. The findings of this research would be published in scientific journal and your name will be kept anonymous. It will bring no consequence to the services that you receive from BRAC or others. So you can be comfortable to respond as you feel. There is no right or wrong answer. We would appreciate if you give us your valuable time in this discussion. Do you agree?

1= Yes 2=No

If the participant agrees take his/her signature and continue with the discussion:

Name and signature of the participants:

S/N	Name	Signature
1.		
2.		
3.		
4.		
5.		
6.		
7.		
8.		

Facilitator: Signature & Date:

Note taker: Signature & Date:

অংশগ্রহনমূলক গবেষণা

PhD project: A capability approach to child growth in haor areas of Bangladesh

সম্মতিপত্রঃ

ভূমিকা

আসসালামুয়ালাইকুম। আমার নাম ....। আমি একটি অংশগ্রহনমূলক গবেষণার কাজে আপনাদের কাছে এসেছি। আমারা মূলত এখানকার দুই বছর এর কম বয়সী বাচ্চারা সুস্থ ভাবে কীভাবে বেড়ে উঠতে পারে তা বোঝার চেষ্টা করছি, যাতে তাদের সমস্যা গুলো চিহ্নিত করে উন্নয়নের জন্য সরকারকে পরামর্শ দিয়ে সাহায্য করতে পারি। এ গবেষণা কার্যক্রমটি নেদারল্যান্ড এর Groningen University, BRAC School of Public Health, BRAC University এবং LANSA এর যৌথ উদ্যোগে, DFID এবং Eric Bleumink Fund (EBF) এর সহযোগিতায় পরিচালিত হচ্ছে। এ কার্যক্রমের শুরুতে আমরা বিভিন্ন হাওর এলাকায় গিয়েছি এবং সেখানকার বাচ্চাদের মা ও বাবাদের সাথে কথা বলেছি। এখানকার ঋতু ও পরিবেশ সবকিছু দেখে এখানকার সমস্যা গুলো বোঝার চেষ্টা করেছি। এখন সেই তথ্যসমূহ আমরা আরেকবার আপনাদের কাছ থেকে যাচাই করে নিতে চাই যেন আমরা সরকার ও ব্র্যাক বা অন্য যারা আপনাদের জন্য কাজ করছে তাদের সঠিক তথ্য দিয়ে সাহায্য করতে পারি।

এই আলোচনা সম্পন্ন করতে ৫০–৬০ মিনিট সময় লাগতে পারে। এ আলোচনায় বোঝার সুবিধার জন্য কিছু ছবি ব্যাবহার করা হবে। আপনাদের সদয় অনুমতি সাপেক্ষে এই আলোচনা থেকে প্রাপ্ত তথ্যসমূহ আমরা লিখে নিব এবং রেকর্ড করবো। এ আলোচনায় অংশগ্রহণ করা বা না করা সম্পূর্ণ আপনাদের ইচ্ছাধীন। আপনারা যেকোনো সময় কোন কারন প্রদর্শন ছাড়াই এ আলোচনা বন্ধ করতে পারেন। তথাপি আপনাদের মতামত ও দৃষ্টিভঙ্গি আমাদের কাছে খুবই গুরুত্বপূর্ণ। এক্ষেত্রে সঠিক বা ভুল উত্তরের কোন বিষয় নেই। আপনারা কোন প্রশ্নের উত্তর দিতে সংকোচ বোধ করলে আমাকে জানাতে পারেন এবং প্রশ্নের উত্তর না ও দিতে পারেন। আলোচনা চলাকালীন সময়ে আপনাদের একজন করে কথা বলার জন্য অনুরোধ করছি যেন আপনাদের কথাগুলো ভালোভাবে রেকর্ড করা যায়।

এখানে আমরা কোন সেবা বা পরামর্শ বা আর্থিক সুবিধা (টাকা–পয়সা) দিতে আসি নি। তবে এ আলোচনা থেকে প্রাপ্ত তথ্য আপনাদের অবস্থার উন্নয়নের লক্ষ্যে কর্তৃপক্ষকে জানানোর ক্ষেত্রে সাহায্য করবে। এ গবেষণার ফলাফল শুধুমাত্র বৈজ্ঞানিক কাজের জন্য প্রকাশ করা হবে এবং আপনাদের নাম কোথাও প্রকাশ করা হবে না। এ আলোচনায় অংশগ্রহনের ফলে আপনার ব্যাক্তিগত জীবনে অথবা ব্র্যাক বা অন্য কোন সংগঠন থেকে প্রাপ্ত সেবার উপর কোন খারাপ প্রভাব পড়বে না। আপনি নির্দ্বিধায় এই আলোচনায় অংশগ্রহণ করে আপনার মূল্যবান মতামত জানাতে পারেন। আপনার এ অংশগ্রহন আমাদের সকলের কাছে প্রশংসিত হবে। আপনি কি এই দলীয় আলোচনায় অংশগ্রহন করতে আগ্রহী?

১=হ্যাঁ ২=না

অংশগ্রহণকারী রাজী হলে তার স্বাক্ষর নিয়ে আলোচনা শুরু করুন।

অংশগ্রহণকারীর নাম ও স্বাক্ষরঃ

ক্রমিক নং	নাম	স্বাক্ষর
১		
২		
৩		
৪		
৫		
৬		
৭		
৮		

Facilitator: Signature & Date:

Note taker: Signature & Date:

উপজেলাঃ গ্রামঃ

পরিচিতি মূলক প্রশ্নঃ

প্রথমেই আমরা পরিচিত হয়ে নেই, আপনারা একের পর এক আপনাদের সম্পর্কে বলুন৷ আপনাদের, পেশা, শিক্ষা, ছেলেমেয়েদের সম্পর্কে বলুন৷

As an introduction, let's go around so that you can introduce yourselves, and tell us about your occupation, education, and children.（Note the background information into the table）

অংশগ্রহণকারীদের বিস্তারিত（Participants' details）:

	বয়স Age	পেশা Occupation	শিক্ষা Education	শিশুর সাথে সম্পর্ক Relationship with child	সন্তানের সংখ্যা Number of Children		২ বছরের কম বয়সী সন্তানের সংখ্যা Number of children <2 years	সব থেকে ছোট সন্তানের বয়স Age of youngest child
					জীবিত Alive	মৃত lost		
1								
2								
3								
4								
5								
6								
7								
8								

A) General question

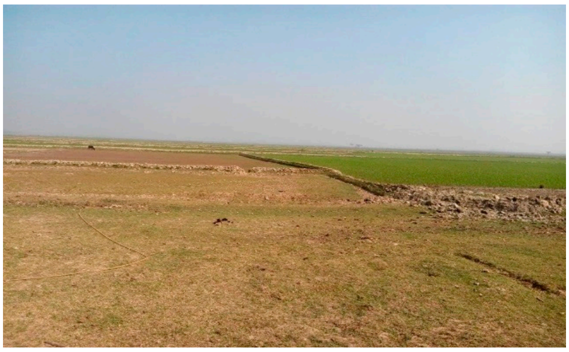 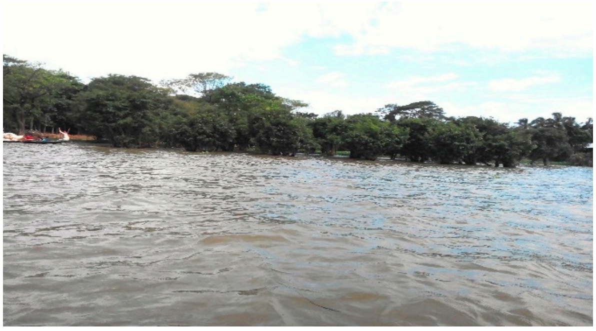

Pics: 1) dry season, 2) wet season (these pictures were captured during the first round of data collection)

We know that the seasonal context in haor is different in comparison to other areas of Bangladesh, for example, the areas remain flooded about half of the year, and then gradually the water recedes and the areas get dry. Now we will show you some pictures given the seasonal context of haor.

আমরা জানি যে এই অঞ্চলে বছরের বেশ লম্বা সময় বন্যার পানি থাকে এবং বাকি সময় শুকনা মৌসুম থাকে।এ প্রসঙ্গে আমি আপনাদের এখন কয়েকটি ছবি দেখাব।

a) The first picture represents the dry season in haor areas. What kind of challenges do people go through in maintaining their life during this period? If people mention about any challenge asks, why does it happen? How does it affect people's daily life? What about children? What kind of supports do people need to address such challenges? And who can help people to address the needs and how?
প্রথম ছবিটি হাওরের শুকনো মৌসুম এর ছবি। এরকম সময়ে এই এলাকার লোকজন প্রতিদিন কী কী ধরনের অসুবিধার মধ্য দিয়ে যেতে হয়? যদি তারা কোন অসুবিধার কথা বলে থাকেন, জিজ্ঞেস করুন কেন এরকম হয়? এতে তাদের শিশুদের কী কী সমস্যা হয়? এই সমস্যাগুলো মোকাবিলা করতে কি ধরনের সহযোগিতা প্রয়োজন? এবং কারা এই কাজে তাদের সাহায্য করতে পারে ও কিভাবে?

b) The second picture represents the wet season in haor areas. What kind of challenges do people go through during this period? Why does it happen? How does it affect people's life? What about children? What kind of supports do people need to address those challenges? And who can help people to address their needs and how?
দ্বিতীয় ছবিটিতে আমরা বন্যার সময়ের দৃশ্য দেখতে পাচ্ছি। এরকম সময়ে এই এলাকার লোকজন প্রতিদিন কী কী ধরনের অসুবিধার মধ্য দিয়ে যেতে হয়? যদি তারা কোন অসুবিধার কথা বলে থাকেন, জিজ্ঞেস করুন কেন এরকম হয়? এতে তাদের শিশুদের কি কি সমস্যা হয়? এই সমস্যাগুলো মোকাবিলা করতে কী কী সহযোগিতা প্রয়োজন?

c) During the transition phases from dry to wet or wet to dry seasons. What do people go through during this period? If people mention about any challenge asks, how do people see it? How does it affect people's daily life? What about the children? What kind of supports do people need to address the challenges they face? And who can help people to address their needs and how?

যখন বন্যা বা খরা কোনটাই নয়, মাঝামাঝি একটা অবস্থা, এরকম সময়ে এই এলাকার লোকজন কি কি ধরনের অসুবিধার মধ্য দিয়ে যেতে হয়? যদি তারা কোন অসুবিধার কথা বলে থাকেন, জিজ্ঞেস করুন কেন এরকম হয়? এতে তাদের শিশুদের কী কী সমস্যা হয়? এই সমস্যাগুলো মোকাবিলা করতে কী কী সহযোগিতা প্রয়োজন?

B) Specific questions on capabilities

Children's capabilities

1. Being able to stay away from disease and eat well-(ask the following questions)

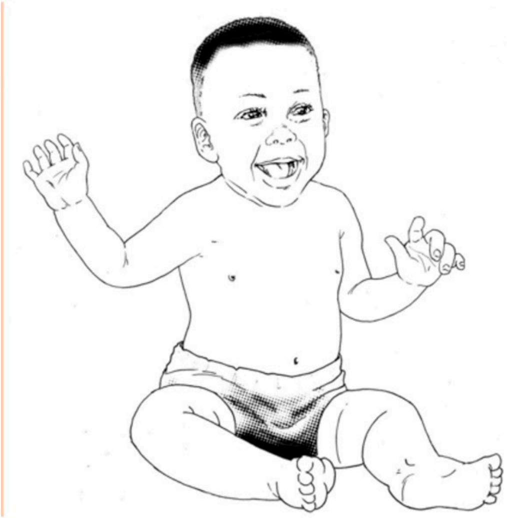
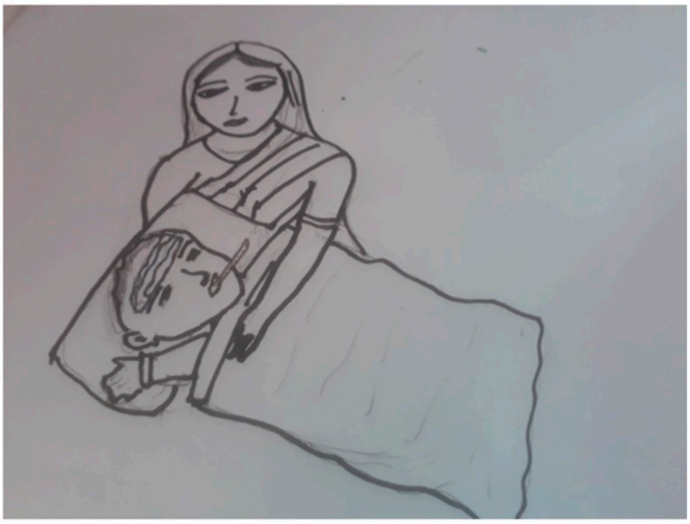

Pic 4: healthy baby (sourced from: https://iycf.spring-nutrition.org/content/people-healthy-baby-6-24-mo-00a-non-country-specific, Pic 5: Sick baby (drawing)

Question: Here we see two pictures. In the 1st picture we see a healthy and smiling baby. In the second picture, we see a bit different, a sick baby with his or her mother who is sitting beside him to provide care. Now we already discussed about different seasons in haor, like sometimes the areas remain wet, and sometimes dry. In such situation, what kind of challenges do the children face in staying away from disease? (Probe: wet season, dry season, transition phase)? What do the parents go though in ensuring their children are able to stay away from disease? How these challenges could be addressed? Who can help them in

addressing the challenges and how (probe: family, community, govt. and institutions like BRAC)?

এখানে আমরা দুটি ছবি দেখতে পাচ্ছি। প্রথম ছবিটি একটি সুস্থ ও হাশিখুশি শিশুর ছবি। অন্যটিতে একটি অসুস্থ শিশু শুয়ে রয়েছে। তার মা বা এরকম কেউ তার পাশে বসে তার যত্ন নিচ্ছে। এখন হাওর এর মত একটি অঞ্চলে যেখানে বছরের বেশিরভাগ সময় পানি থাকে, বা কখনো শুকনা থাকে সেরকম অবস্থায় একটি শিশু সুস্থ থাকার জন্য শিশুটিকে কি কি সমস্যার মুখোমুখি হতে হয়? ? (probe: বর্ষা কালে বা শুকনা মৌসুমে, বা অন্য মাঝামাঝি সময়ে)? শিশুর মা বাবা কে কী ধরনের সমস্যা মোকাবিলা করতে হয়?এই সমস্যাগুলো ঠিক করার জন্য শিশুটির ও তার বাবা মায়ের বা যারা তার যত্ন নেয় তাদের কি ধরনের সহযোগিতা দরকার?কারা তাদের এ সহযোগিতা করতে পারে এবং কিভাবে? (Probe করুনঃ এলেকার লোকজন,সরকারী/বেসরকারি সংস্থা, ব্র্যাক)

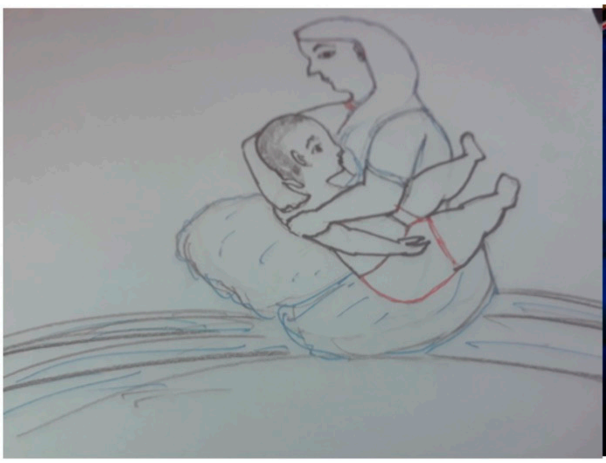

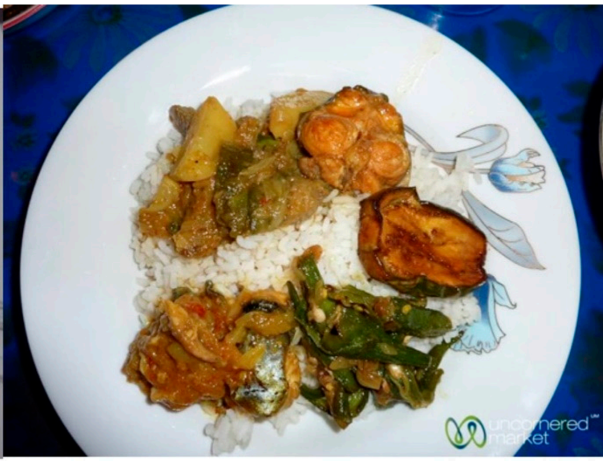

Pic 6: Mother is breastfeeding her child (drawing), Pic 7: Food plate (sourced from: https://uncorneredmarket.com/bangladeshi-food/)

Question: Here we see a mother is breastfeeding her baby and the other picture shows diverse food on the plate. In our last visit people said that the children to be able to grow well are required to be able to eat well or to be fed well. Now how do people define whether a child is eating well or not? What do the children need to be able to eat well? What kind of challenges do the children endure to eat well? How does it influence their growth? What do their parents need to feed them well? What kind of challenges do they go through to feed them well? How better the unmet needs can be addressed (discuss at different level as arises during discussion such as parents level, community level, institutional level)?

এখানে আমরা দেখছি একটি মা তার শিশুকে বুকের দুধ খাওয়াচ্ছে, আরেকটি ছবিতে আমরা থালা ভরা খাবার দেখতে পাচ্ছি।এর আগে আমরা যখন এসেছিলাম তখন এখানকার মা ও বাবারা বলেছিল যে একটি শিশু কে ভালো মত বেড়ে উঠতে গেলে ভালমতো খেতে হবে। এই ভালমতো খেতে হবে বলতে আসলে এই এলাকায় কি বুঝায়? শিশু যেন ভালোমতো খেতে পারে এজন্য কী কী দরকার? এক্ষেত্রে শিশু ও শিশুর বাবা মা কে কী ধরনের সমস্যা মোকাবেলা করতে হয়? (probe: বর্ষা কালে বা শুকনা মৌসুমে, বা অন্য মাঝামাঝি সময়ে)। এ সমস্যা মোকাবেলা করতে কী ধরনের সহযোগিতা দরকার? কারা তাদের এ সহযোগিতা করতে পারে এবং কিভাবে? (Probe করুনঃ মা বাবা, পরিবার, এলাকার লোকজন, সরকারী/বেসরকারি সংস্থা, ব্র্যাক)

2. Being able to be born with God’s blessings and the hereditary traits needed to grow in size- (ask the following questions)

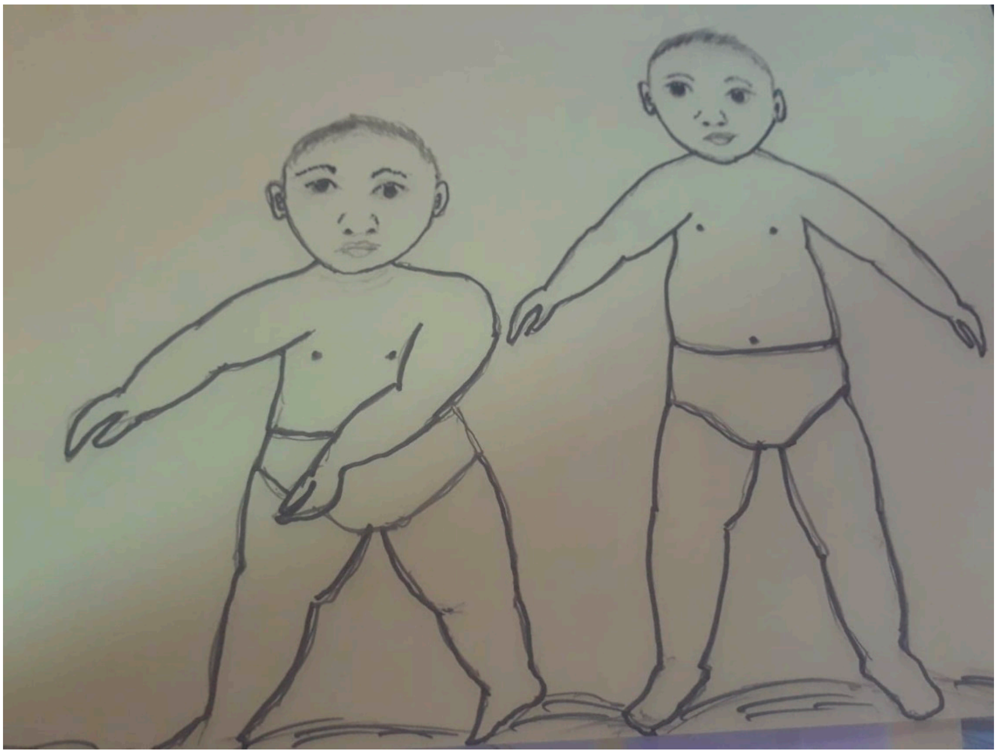

Pic 8: Tall and short baby (drawing)

Question: As we see in the picture, there are two children, one is tall and the other is short, how do the people in haor see this difference? Why does it happen in haor? What do the children of haor need to have to be able to grow in size in this area (probe: God’s will or family history)? What are the challenges they endure in growing in size? Who can help if a

child is not able to grow in size and how (Probe at different levels, parents, communities, Govt. and BRAC)?

এই ছবিতে আমরা দেখতে পাচ্ছি একটি শিশু লম্বা এবং অন্যজন খাটো।এই বিষয়টাকে এখানকার লোকজন কিভাবে দেখে? এরকম কেন হয়?একটি শিশুর শারিরিক বৃদ্ধির জন্য বা লম্বা হতে হলে কি কি দরকার (Probe করুনঃ আল্লাহর ইচ্ছা, বংশের ধারা)। এ ক্ষেত্রে তাদের কী কী সমস্যা হয়? এ সমস্যা কিভাবে দূর করা যায়? কারা এ ক্ষেত্রে সহযোগিতা করতে পারে এবং কিভাবে? (Probe করুনঃ মা বাবা, পরিবার, এলাকার লোকজন, সরকারী/বেসরকারি সংস্থা, ব্র্যাক)

3. Being able to stay happy and playful- (ask the following questions)

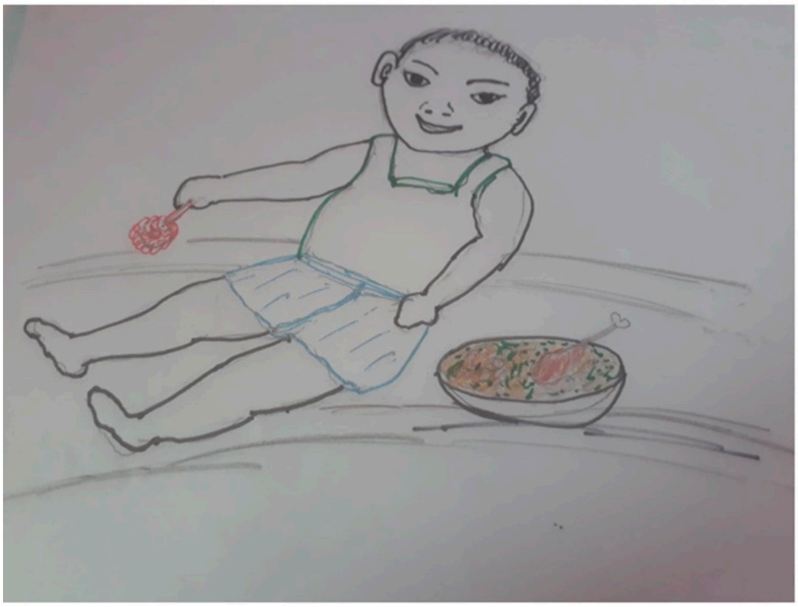

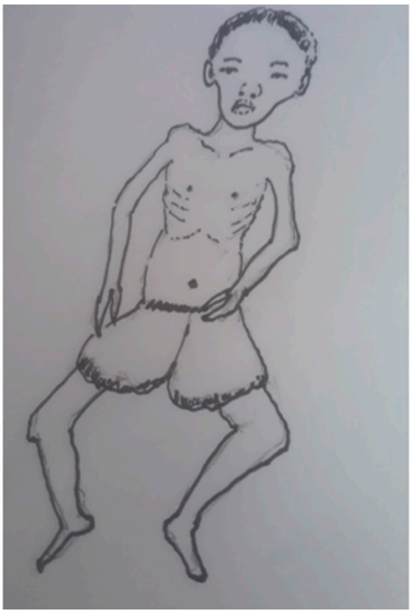

Pic 9: a well-nourished smiling, playful baby (drawing), Pic 10: a malnourished sad baby (drawing)

Question: We see two babies in the pictures, one looks happy, well-nourished and holding something in his hand (he is probably playing with it), and the other looks sad, thin or undernourished. How do people in haor see/ value such difference? Why does it happen? What do the babies of haor need to have to be able to stay happy and playful? What kind of challenges do they endure? What kind of support do they need to overcome the challenges? Who can play important role in this process and how? (Discuss at different level, parents, family, community, and institutional level: Govt. NGOs, BRAC)

এখানে আমরা দেখতে পাচ্ছি, একটি শিশু বেশ হাশিখুশি, সাস্থ্য ও পুষ্টি ভাল, হাতে কিছু একটা নিয়ে খেলছে। অন্য শিশুটি একটু রোগা, মন খারাপ বা অপুষ্টিতে ভুগছে। এই বিষয়টাকে এখানকার লোকজন কিভাবে দেখে? এরকম কেন হয়? হাওর অঞ্চলে একটি শিশু যেন হাশিখুশি থাকে, বা স্বাভাবিক খেলাধুলা করে সেজন্য কি কি দরকার? এ ক্ষেত্রে শিশুরা কি ধরনের বাধা বিপত্তির সম্মুখীন হয়? এই

বাধাবিপত্তি অতিক্রম করার ক্ষেত্রে তাদের কি ধরনের সহযোগিতা দরকার? কে বা কারা তাদের কিভাবে এই সহযোগিতা করতে পারে? (Probe করুনঃ মা বাবা, পরিবার, এলাকার লোকজন, সরকারী/বেসরকারি সংস্থা, ব্র্যাক)

Maternal Capabilities

1. Being able to stay healthy and eat well-(ask the following questions)

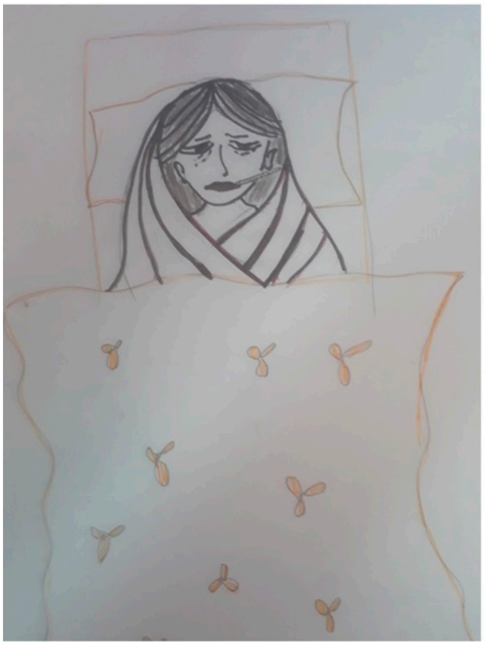
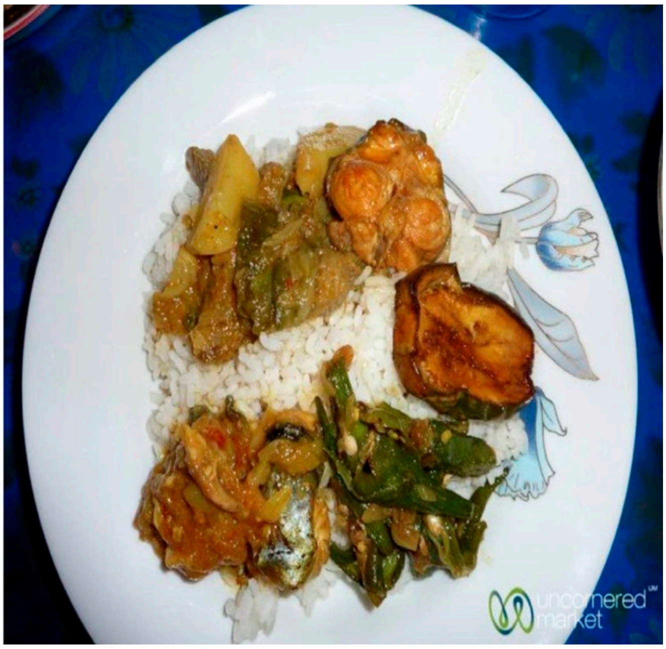

Pic 11: a sick mother (drawing), Pic 12: diverse foods on the plate (sourced from: https://uncorneredmarket.com/bangladeshi-food/)

Question:

a) In the first picture, we see a sick mother lying on the bed. If a mother gets sick what happens? What happens to the child? What do the mothers need in haor to avoid sickness and stay healthy? How the family, communities, BRAC and Govt. can help them to address their needs so that they can stay healthy?

এই ছবিতে আমরা একজন অসুস্থ মা কে দেখতে পাচ্ছি। যদি একজন মা অসুস্থ হয়ে যায় তাহলে কী কী সমস্যা হয়? এ ক্ষেত্রে তার বাচ্চার কী সমস্যা হয়? মা যেন সুস্থ থাকে বা অসুস্থ হয়ে না পরে সেজন্য তার কী কী সুবিধা বা সহযোগিতা দরকার? কে বা কারা তাদের কিভাবে এই সহযোগিতা করতে পারে? (Probe করুনঃ মা বাবা, পরিবার, এলাকার লোকজন, সরকারী/বেসরকারি সংস্থা, ব্র্যাক)

b) In the other picture, we see there are diverse foods on the plate. Last time some parents told us that mother need to be able to eat well for their own energy or to be able to feed their children. How do people define if a mother is eating well, based on

which conditions or indicators (probe: pregnancy period, lactation)? And why? How does it relate to their baby's growth? What kind of challenges do the mothers face in 'eating well' at different seasons? How do people address these challenges or how these challenges might be solved or addressed? (Probe at different levels:family, community, institutional)

এই ছবিতে দেখা যাচ্ছে থালা ভরা নানারকম খাবার। এর আগে যখন আমরা এসেছিলাম তখন বাচ্চার মা বাবারা বলেছিল যে মায়ের ভাল ভাল খাবার খেতে হবে যেন তারা নিজেরা শক্তি পায় এবং বাচ্চাকে খাওয়াতে পারে। এই ভাল খাওয়া বলতে আসলে এখানে কি বুঝায়? কি ধরনের খাওয়া মায়েদেরকে খেতে বলা হয় এবং কেন? (Probe করুনঃ গর্ভবতী অবস্থা, সন্তান প্রসব পরবর্তি সময়) । এই ভাল খাবার খেতে মায়েরা কী কী ধরনের বাধা বিপত্তি হয়? এই বাধা বিপত্তি মোকাবিলা করতে তাদের কী ধরনের সহযোগিতা দরকার? কে বা কারা তাদের কিভাবে এই সহযোগিতা করতে পারে? (Probe করুনঃ মা বাবা, পরিবার, এলাকার লোকজন, সরকারী/বেসরকারি সংস্থা, ব্র্যাক)

2) Being able to stay away from domestic violence

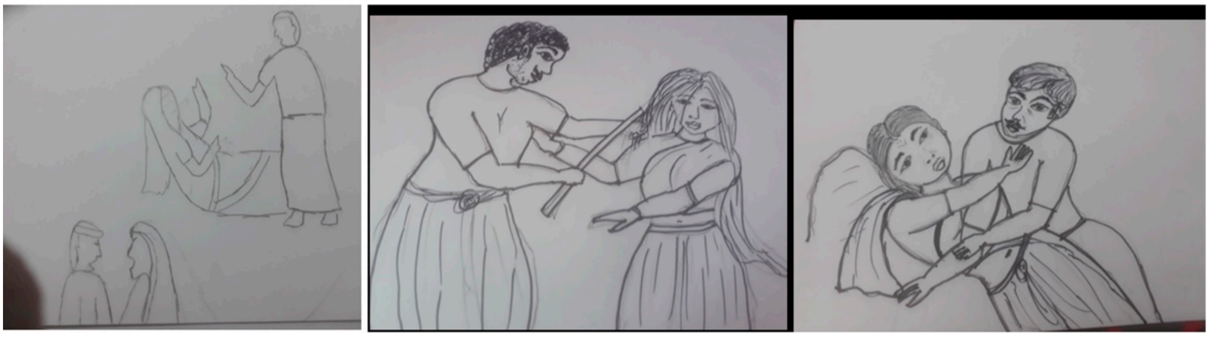

Pics 13: Couples' clashes at different situations (drawings)

Question: From the pictures, we see the couples are having clashes at different situations, and at every situation the husband is trying to convince/force his wife through yelling, or beating. In haor what happens to the women in such situations? How does it affect their young child? What do women need to avoid/resist this? Who can help them to resist and how (Probe at individual, family, community, and institutional level)

এই ছবিগুলিতে আমরা দেখতে পাচ্ছি যে স্বামী ও স্ত্রীর মধ্যে ঝগড়া হচ্ছে এবং স্বামী তার স্ত্রীকে শারিরিকভাবে আঘাত করছে বা বল প্রয়োগ করে কিছু একটা বুঝানোর চেষ্টা করছে।হাওর এলাকায় এরকম হলে মানুষ বিষয়টাকে কিভাবে নেয়? এয় অবস্থা যাতে না হয় মায়েরা কি করে? বা হলে কিভাবে প্রতিরোধ করে? এ ক্ষেত্রে তাদের ক বা কারা কিভাবে সাহায্য করতে পারে? (Probe করুনঃ মা বাবা, পরিবার, এলাকার লোকজন, সরকারী/বেসরকারি সংস্থা, ব্র্যাক)

3) Being able to allocate time for child care as desired

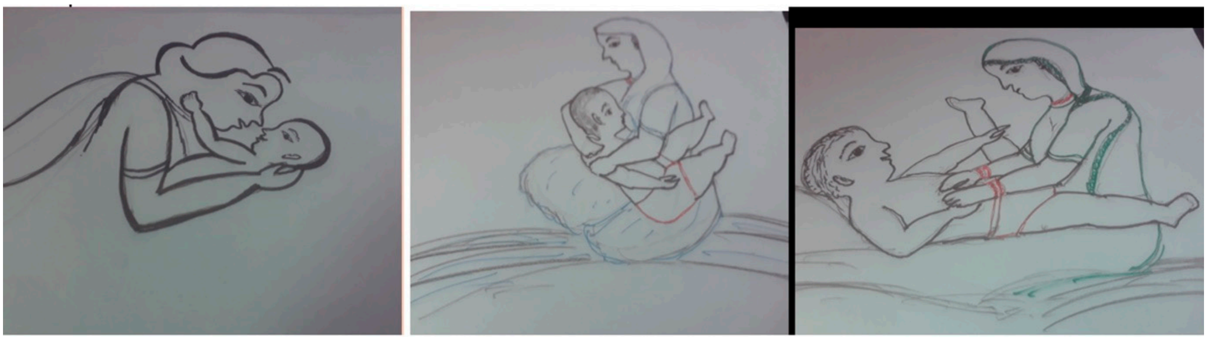

Pic 14: A mother is playing (through expressing love) with her baby, Pic 15: A mother is feeding her baby, Pic 16: A mother is massaging her baby (drawings)

Question: In the pictures, we see a mother is playing (through expressing love) with her baby, breastfeeding her baby or massaging her baby with oil. In haor what kind of care do the mothers provide to their children at early age (below 2 years of age) so that they can grow well? How they manage time for their child care after doing their other work (household chores)? What kind of struggles do they face to allocate time for child care according to their own desire? What do they need to allocate adequate time for child care according to their own desire? What might be the possible ways to address their needs (Probe at individual, community and institutional level)?

এই ছবিগুলোতে আমরা দেখতে পাচ্ছি মা তার বাচ্চার সাথে খেলছে, বা বাচ্চাকে খাওয়াচ্ছে, বা তেল দিয়ে মালিশ করছে। হাওরে ২ বছরের কম বয়সী শিশুদের ঠিকমত বেড়ে উঠার জন্য মায়েরা কী ধরনের যত্ন নিয়ে থাকে? ঘরের বা অন্যান্য কাজকর্ম করে তারা এ জন্য কিভাবে সময় বের করে? নিজের মত করে এ সময় বের করতে তাদের কি কি সমস্যা পোহাতে হয়? এ সমস্যা দূর করতে হলে তাদের কি কি দরকার? বা কী ধরনের সহযোগিতা দরকার? কে বা কারা তাদের কিভাবে এই সহযোগিতা করতে পারে?

(Probe করুনঃ মা বাবা, পরিবার, এলাকার লোকজন, সরকারী/বেসরকারি সংস্থা, ব্র্যাক)

4) Being able to be engaged in income- generating activities

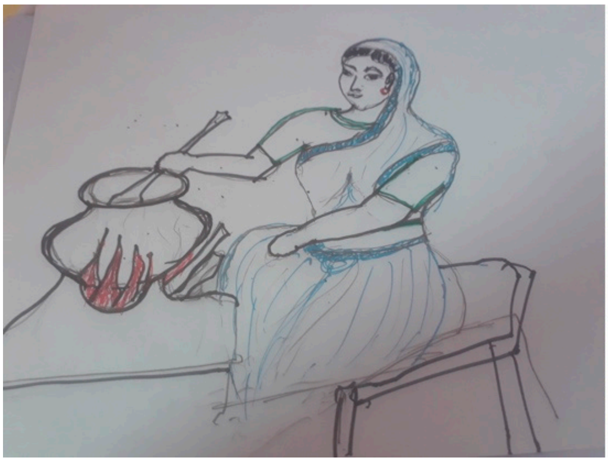
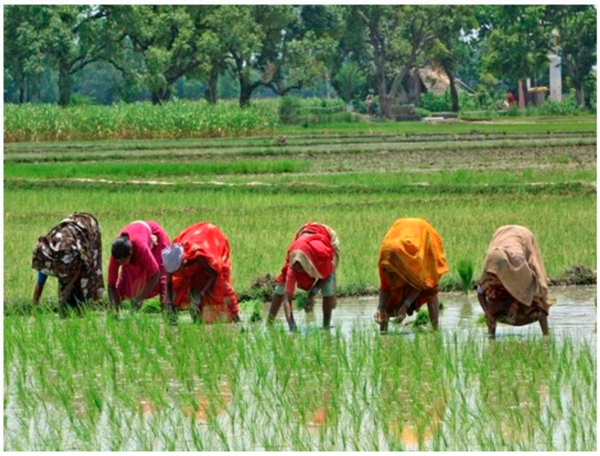

Pic 18: A mother is cooking (drawing), Pic 19: women are working in the paddy field (source: https://www.shethepeople.tv/news/women-farmers-in-india-where-they-stand/)

Question: Here we see women are engaged in different activities such as cooking, field work, in addition to their child care activities as we saw in haor last time. What happens in haor areas? Do they do any other works in addition to the activities I mentioned? Do they do any paid job? When most of the women are engaged with unpaid job, how do they access money when they need? How does it influence their children (probe: child care activities, physical growth, and mental development)? If they mention about any problem ask how that can be solved? What do they need to be able to earn or access money? Who can support them to address their need in earning/accessing money (Probe: individual level, family, community or institutional)

এই ছবিতে আমরা দেখতে পাচ্ছি মায়েরা শিশুর লালন পালন ছাড়াও বিভিন্ন ধরনের কাজে ব্যস্ত আছেন যেমন, রান্না করা, মাঠে কাজ করা। এবং এজন্য তারা কোন অর্থ আয় করেনা। এই অঞ্চলে মায়েরা এ ছাড়া আর কি কাজ করে থাকে? তারা কি অর্থ আয় করা যায় এরকম কোন কাজ করে থাকে? যখন মায়েরা কোন রোজগার করে না তারা কিভাবে অর্থ পেয়ে থাকেন? এরকম ক্ষেত্রে তাদের বাচ্চাদের উপর কী ধরনের প্রভাব পরে (*Probe* করুনঃ শিশুর যত্ন, শারীরিক/মানসিক বৃদ্ধি বা বিকাশ)? কোন সমস্যার কথা উল্লেখ করলে জিজ্ঞেস করুন কিভাবে ওই সমস্যার সমাধান করা যায়? অর্থ রোজগার করার জন্য মায়েদের কী ধরনের সহযোগিতা দরকার? কে বা কারা তাদের কিভাবে এই সহযোগিতা করতে পারে? (*Probe* করুনঃ পরিবার, এলাকার লোকজন, সরকারী/বেসরকারি সংস্থা, ব্র্যাক)?

5) Being able to express love (maya-mohabbat) and take care of the children

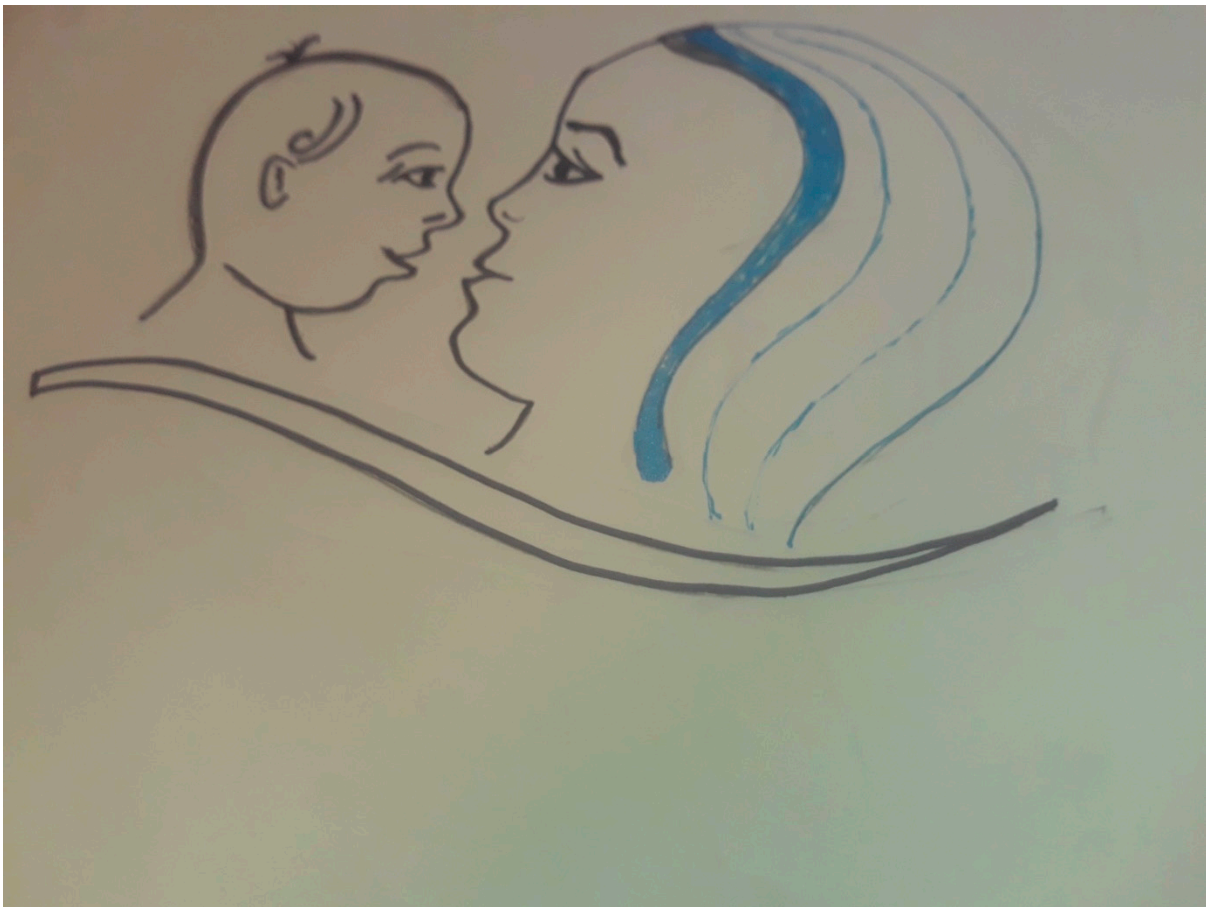

Pic 20: A mother is expressing her love to her child (drawing),
Question: In the pictures, we see a mother is expressing her love to her child. In haor what does a mother need to have to be able to express her love for her child? How does it stimulate a child's growth/ development? What kind of challenges does a mother face in expressing her love to the child? How a mother can be supported in addressing the challenges to make her capable of expressing love to her child as much as she desires? (Family, Communities, Govt., Institutions such as BRAC)

এই ছবিতে আমরা দেখতে পাচ্ছি একজন মা তার বাচ্চাকে আদর করছে। এর আগেরবার যখন হাওর অঞ্চলে এসেছিলাম তখন অনেকে বলেছিল মায়েরা তাদের বাচ্চাকে আদর ভালবাসা দিতে হবে তাহলে বাচ্চার ভাল হবে।এই আদর ভালবাসা বাচ্চার কী ধরনের ভাল করে থাকে? (*Probe* করুনঃ শারীরিক মানসিক বৃদ্ধি বা বিকাশ) এ ভালবাসা প্রকাশ করার ক্ষেত্রে মায়েদের কী কী বাধা বিপত্তি আসে?

এই বাধা বিপত্তি দূর করতে তাদের কি ধরনের সহযোগিতা দরকার? কে বা কারা তাদের কিভাবে এই সহযোগিতা করতে পারে?

(*Probe* করুনঃ পরিবার, এলাকার লোকজন, সরকারী/বেসরকারি সংস্থা, ব্র্যাক)

6) Being able to be educated in order to provide the children with good care

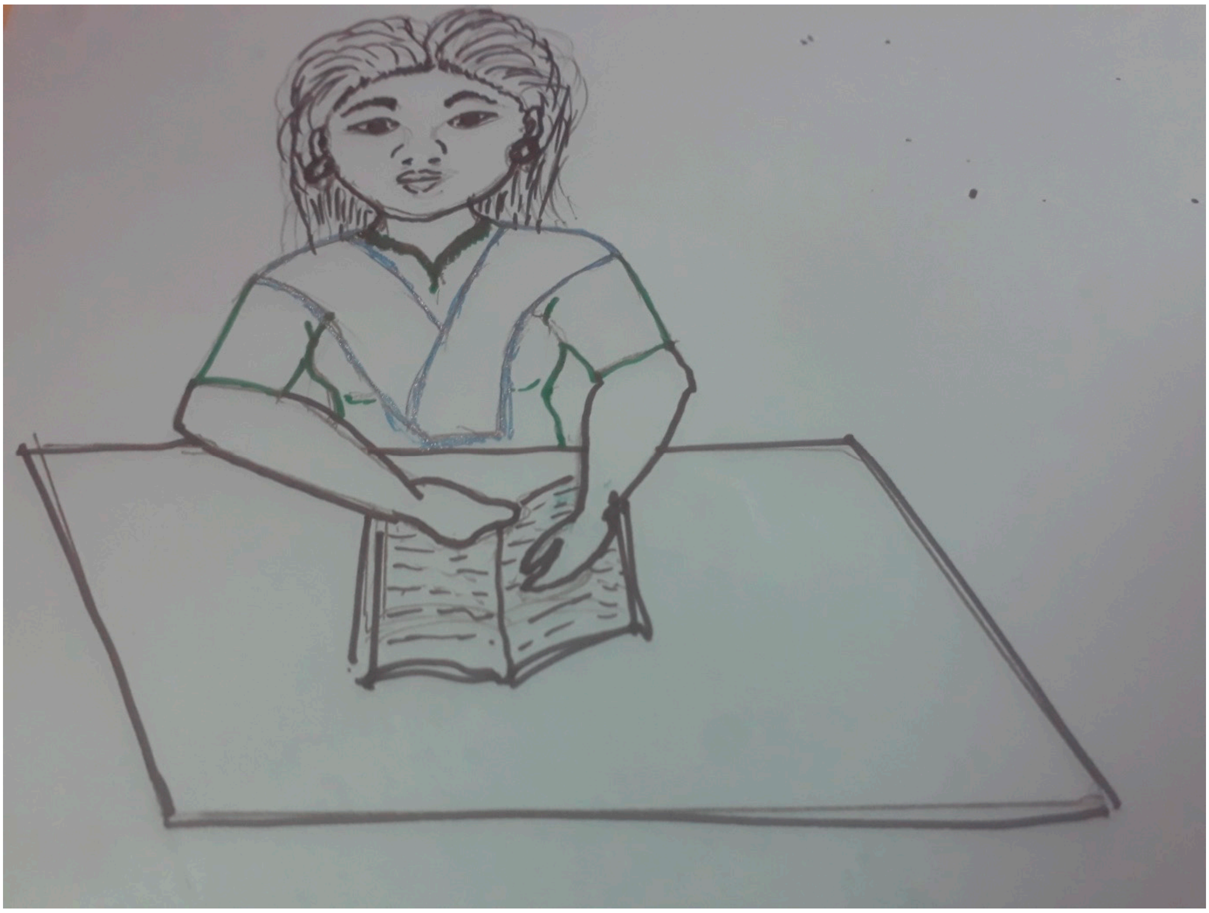

Pic 21: A girl is studying (drawing)

Question: We see a girl with a book and she has been studying. It is likely that in future this girl will grow up as a mother. How do you see the significance of receiving education for a mother in context of how a child grows in haor? What are the challenges do the mothers endure to be educated? How a mother can be supported in addressing the challenges to become educated (Probe: family, communities, Govt. and NGOs like BRAC)?

আমরা দেখতে পাচ্ছি একটি মেয়ে বই পড়ছে যে ভবিষ্যতে মায়ের ভুমিকা পালন করবে৷ মায়েদের ক্ষেত্রে এরকম লেখাপড়া জানা কতটা জরুরী? মায়ের লেখাপড়া বাচ্চার উপর কিরকম প্রভাব ফেলে (Probe করুনঃ শারীরিক মানসিক বৃদ্ধি বা বিকাশ) I হাওর অঞ্চলে একজন মায়ের শিক্ষিত হতে হলে কি কি বাধা বিপত্তি আসে? এই বাধা বিপত্তি সমাধান করতে হলে তাদের কী ধরনের সহযোগিতা দরকার? কে বা কারা তাদের কিভাবে এই সহযোগিতা করতে পারে? (Probe করুনঃ পরিবার, এলাকার লোকজন, সরকারী/বেসরকারি সংস্থা, ব্র্যাক) ?

<u>Fathers' capabilities</u>

1) Being able to earn in all seasons and provide the family with the things they need

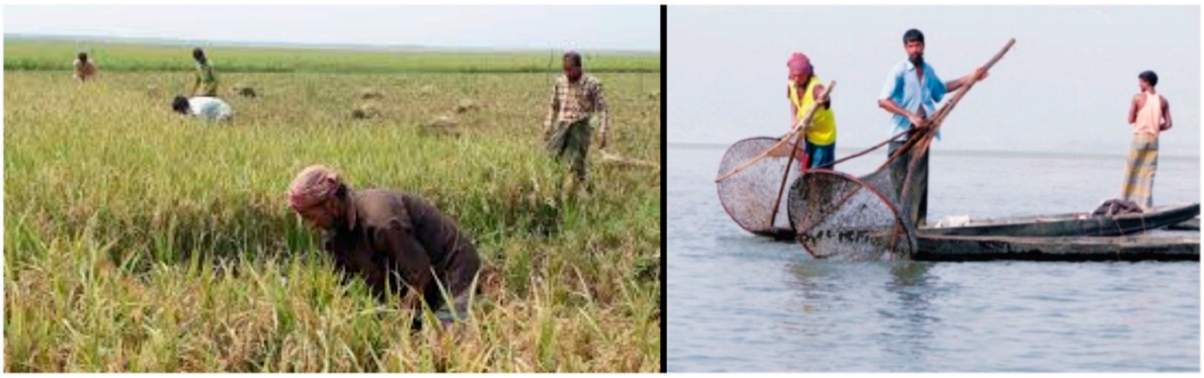

Pic 23: Men are working in field for earning livelihood (pic sourced from: https://www.thedailystar.net/country/bangladesh-water-development-board-asks-sunamganj-farmers-for-quick-harvest-of-paddy-1567120), men are fishing for earning livelihood (images sourced from : https://www.thedailystar.net/starinsight/2010/02/02/cover.htm)

Question: From the pictures, we see the fathers in haor are doing different activities for earning their livelihood. What other activities do men do to earn money at different seasons? How their earning helps them to take care of their children? How does it relate their children's growth? What kind of challenges do they go through in earning money and how that can be addressed? Who can help them in improving their earning and how? (Probe: family, communities, Govt. and NGOs like BRAC)

এই ছবি গুলি তে আমরা দেখতে পাচ্ছি যে হাওর অঞ্চলের বাবারা অর্থ রোজগারের জন্য বিভিন্ন ধরণের কাজ যেমন ধান ক্ষেতের কাজ, মাছ ধরার কাজ ইত্যাদি করছে। এছাড়া আয় করার জন্য বাবারা আর কি ধরণের কাজ করে থাকে? বাবাদের আয় রোজগার শিশুদের কী কাজে লাগে? শিশুদের বেড়ে ওঠার ক্ষেত্রে বাবাদের আয় কিভাবে কাজে লাগে? আয় করতে গিয়ে বাবারা কী কী বাধা বিপত্তির সম্মুখীন হয়ে থাকে (*Probe:* বর্ষা, শুকনা, মাঝামাঝি সময়ে)? এই বাধা বিপত্তি সমাধান করতে হলে তাদের কি ধরনের সহযোগিতা দরকার? কে বা কারা তাদের কিভাবে এই সহযোগিতা করতে পারে? (*Probe* করুনঃ পরিবার, এলাকার লোকজন, সরকারী/বেসরকারি সংস্থা, ব্র্যাক)?

2) Being able to save the future and the one who creates the future

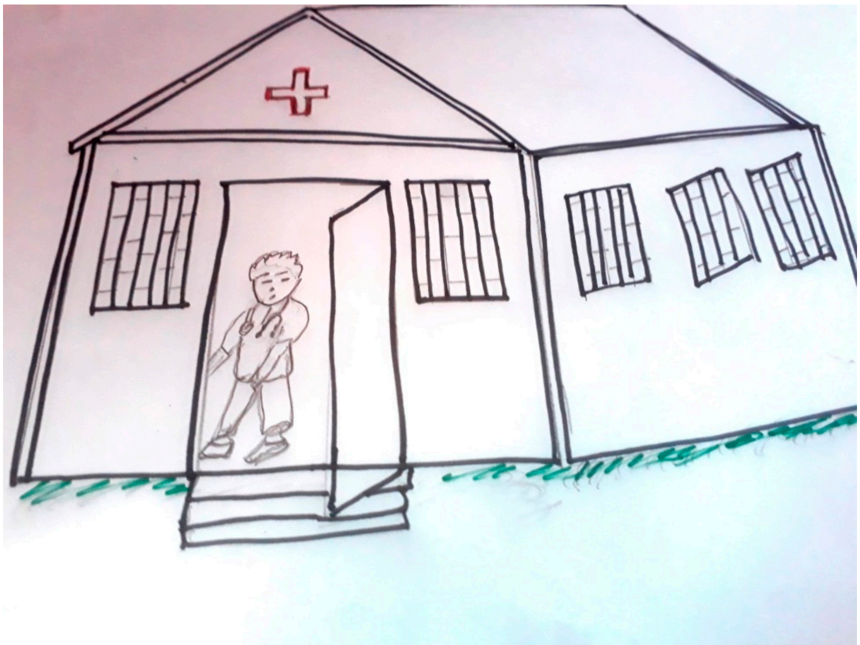

Pic 24: Entrance of a hospital (drawing)

Question: In the above picture, we see a health care facility. In haor how do people reach that place to have a safe delivery? What type of challenges do people endure to ensure a safe delivery (probe: wet season, dry season, transition phases)? What challenges do people face in haor to reach the place (probe: wet season, dry season, transition phases)? How do people handle the challenges? And who can help them in this process and how? (Family, community, govt., other institutions including BRAC)

এই ছবিতে দেখা যাচ্ছে একটি স্বাস্থ্য কেন্দ্রের ছবি। হাওর অঞ্চলে মায়েদের সুস্থভাবে বাচ্চা হওয়ার জন্য মানুষ কি করে থাকে? স্বাস্থ্য কেন্দ্রে পৌঁছাতে হলে মানুষের কি কি বাধা বিপত্তির সম্মুখীন হতে হয় (Probe: বর্ষা, শুকনা, মাঝামাঝি সময়ে)। এই বাধা বিপত্তি তারা কিভাবে মোকাবিলা করে? এর সমাধান করতে হলে তাদের কি ধরনের সহযোগিতা দরকার? কে বা কারা তাদের কিভাবে এই সহযোগিতা করতে পারে? (Probe করুনঃ পরিবার, এলাকার লোকজন, সরকারী/বেসরকারি সংস্থা, ব্র্যাক)?

2. Being able to support their children by bringing them toys or items they need

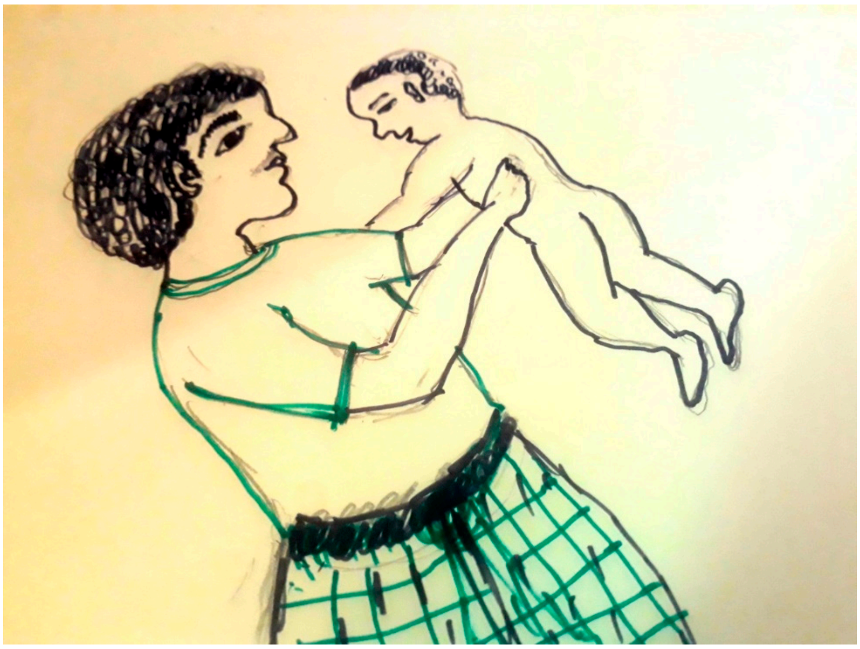

Pic 25: Father with his baby

Question: We see in the picture a father is playing with his baby to make him/her happy. In haor what kind of activities does a father do to make their children happy? To what extent they are able to keep them involved in child care activities and how do people value this? What kind of challenges do they face? How their challenges could be addressed? And who can help them in this process and how? (Probe: family, community, govt., other institutions including BRAC)

এই ছবিতে দেখা যাচ্ছে একজন বাবা তার বাচ্চার সাথে খেলছে তাকে আনন্দ দেবার জন্য।এই অঞ্চলে বাবারা বাচ্চাদের খুশি রাখার জন্য কি করে থাকে? বাচ্চাদের যত্ন নিতে তারা কিভাবে অংশগ্রহণ করে? এই এলাকার লোকজন এয় বিষয়টাকে কিভাবে দেখে? বাচ্চার জন্য এসব করতে বাবার কি কি বাধা বিপত্তির সম্মুখীন হয়ে থাকে? এর সমাধান করতে হলে তাদের কি ধরনের সহযোগিতা দরকার? কে বা কারা তাদের কিভাবে এই সহযোগিতা করতে পারে? *(Probe* করুনঃ পরিবার, এলাকার লোকজন, সরকারী/বেসরকারি সংস্থা, ব্র্যাক) ?

4. Being able to be educated in order to get a job and educate the children

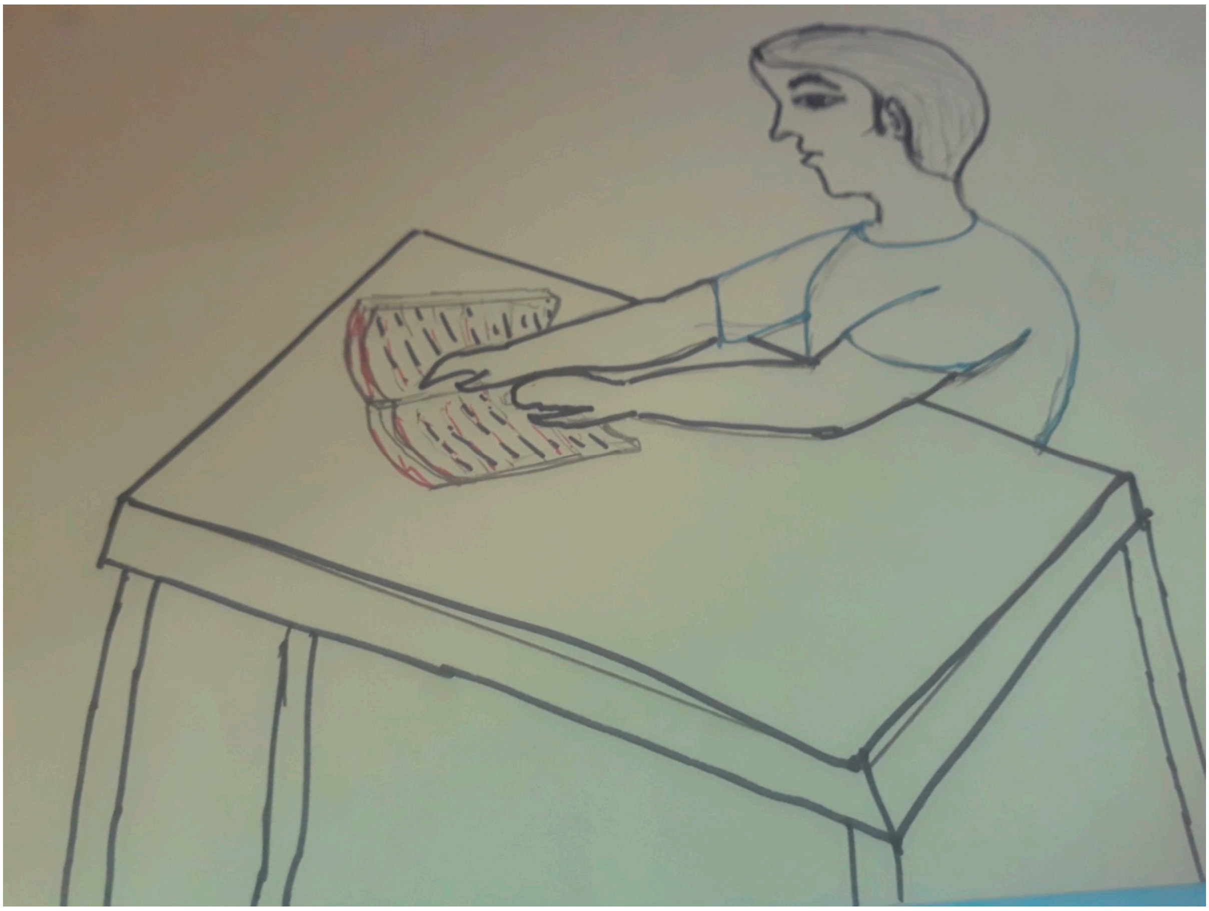

Pic 26: A boy reading book (drawing)

Question: In the picture, we see a boy is studying, who is likely to grow as a father in future. In haor how do people see this? Last time, some parents mentioned that a father may have different capabilities or qualities to provide care to their children, for example, earning, brining toys for the children, and so forth, how education is valued among all those qualities? What kind of challenges do they face? How their challenges could be addressed? And who can help them in this process and how? (Probe: family, community, govt., other institutions including BRAC)

এই ছবিতে দেখা যাচ্ছে যে একটি ছেলে লেখাপড়া করছে যে ভবিষ্যতে বাবার ভুমিকা পালন করতে পারে। এ অঞ্চলে বাবাদের ক্ষেত্রে এরকম লেখাপড়া জানা কতটা জরুরী এবং কেন? বাবার লেখাপড়া বাচ্চার উপর কিভাবে প্রভাব ফেলে (probe: শারীরিক মানসিক বৃদ্ধি বা বিকাশ)। হাওর অঞ্চলে একজন বাবার শিক্ষিত হতে হলে কি কি বাধা বিপত্তি আসে? এই বাধা বিপত্তি সমাধান করতে হলে তাদের কি ধরনের সহযোগিতা দরকার? কে বা কারা তাদের কিভাবে এই সহযোগিতা করতে পারে? (Probe করুনঃ পরিবার, এলাকার লোকজন, সরকারী/বেসরকারি সংস্থা, ব্র্যাক)?

5. Being able to remain in good health, have energy, demonstrate intelligence, and be religious to maintain a family

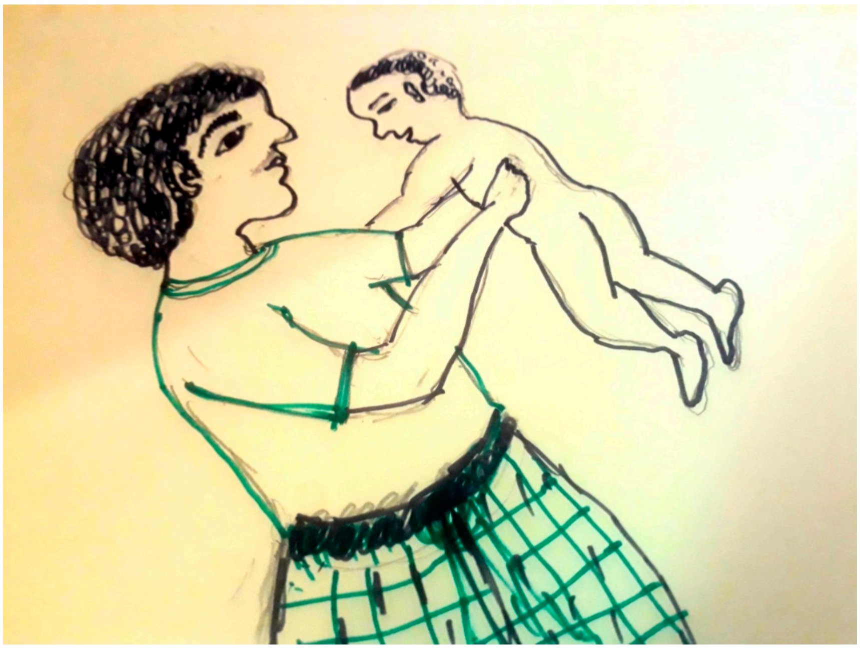

Question: We see a father and a baby in this picture. Some people in haor said a father needs to be healthy, energetic, intelligent and religious to be able to maintain the family in good order. What do you think? Why do they really need to have these aforementioned qualities and how these qualities may influence a child in context of haor? What other qualities or capabilities do they need to have? What do the fathers need to achieve those qualities/capabilities? Who can help them in achieving these qualities and how? (Probe: family, community, govt., other institutions including BRAC)

এই ছবিতে একজন বাবা ও শিশু কে দেখা যাছে। হাওর এর কিছু মানুষ বলেছিল যে একজন বাবার সুস্থ, শক্তিশালি, বুদ্ধিমান ও ধার্মিক হওয়া দরকার যেন তারা পরিবারকে ঠিক মত চালাতে পারে। আপনাদের কি মনে হয়? এ গুনগুলি কেন দরকার? এগুলি বাচ্চার বেড়ে ওঠার জন্য কিভাবে কাজে লাগে? এই গুনগুলো অর্জন করতে বাবার কি কি দরকার এবং কেন? কে বা কারা কিভাবে বাবাদেরকে এই গুনগুলো অর্জন করতে সহযোগিতা করতে পারে? (Probe করুনঃ পরিবার, এলাকার লোকজন, সরকারী/বেসরকারি সংস্থা, ব্র্যাক)?

Household level:

1) Being able to live in a shelter that is safe during the wet season

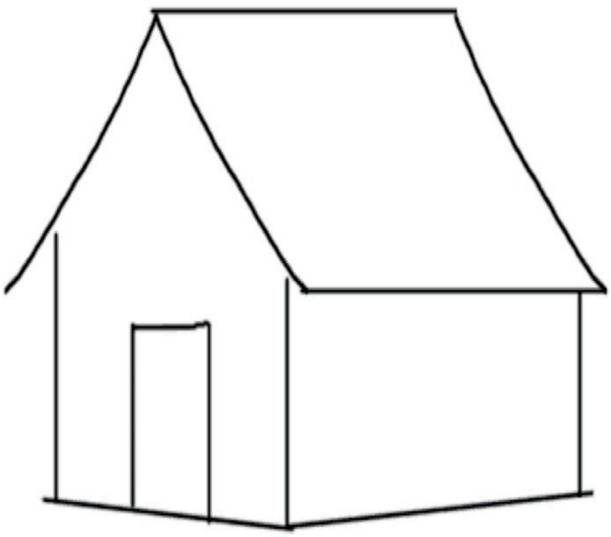

Pic 27: A house (sourced from: https://ya-webdesign.com/imgdownload.html)

Question: We see a picture of a house where people find their shelter. In haor, how do people keep their houses safe for their children at different seasons (probe: wet season, dry season and transition phase)? What kind of challenges do the people in haor face to keep the houses safe and liveable for their growing children? Who can help them in addressing their needs/challenges to keep the shelter safe and how? (Probe: family, community, govt., other institutions including BRAC)

আমরা একটি ঘর দেখতে পাচ্ছি যেখানে মানুষ তার বেঁচে থাকার আশ্রয় খুঁজে নেয়। হাওর অঞ্চলে মানুষ তাদের বাড়িঘর শিশুদের জন্য নিরাপদ রাখার জন্য কি করে থাকে বা কিভাবে নিরাপদ রাখে? (Probe: বর্ষা, শুকনা, মাঝামাঝি সময়ে) এক্ষেত্রে তারা কি কি বাধা বিপত্তির সম্মুখীন হয়? শিশুদের জন্য বাড়ি নিরাপদ রাখতে কি কি দরকার হয়? এই বাধা বিপত্তি সমাধান করতে হলে তাদের কি ধরনের সহযোগিতা দরকার? কে বা কারা তাদের কিভাবে এই সহযোগিতা করতে পারে? (Probe করুনঃ পরিবার, এলাকার লোকজন, সরকারী/বেসরকারি সংস্থা, ব্র্যাক)?

3. Being able to overcome struggles with the earth and to keep the child neat and clean

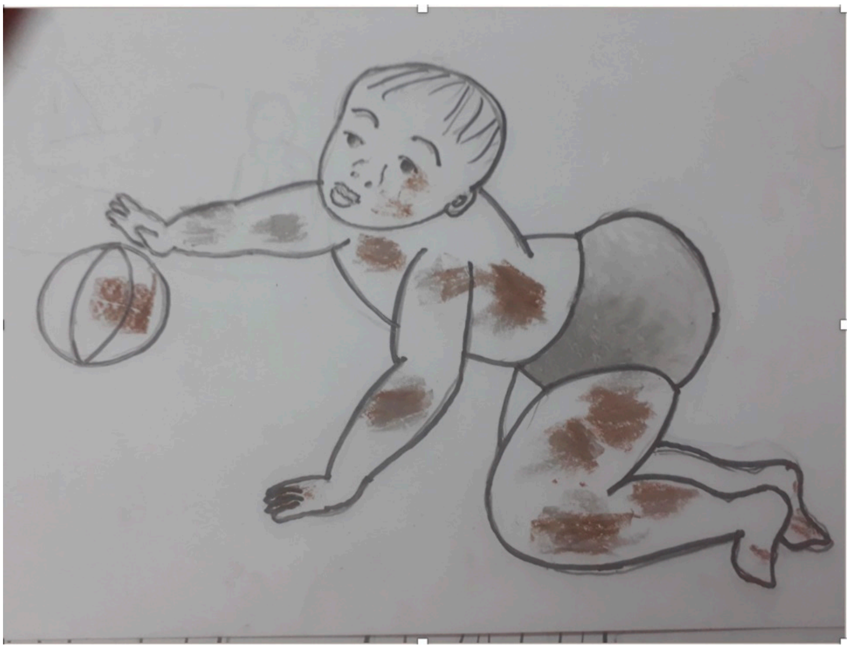

Pic 28: A baby covered with dirt/muds (drawing)

Question: We see that a baby is covered my muds and dirt. When it happens to the children in haor how do the parents keep them clean? What kind of challenges do they face in keeping them neat and clean? How their challenges can be overcome and who can help them in doing so (Probe: family, community, govt., other institutions including BRAC)

এই ছবিতে দেখা যাচ্ছে একটি শিশুর গায়ে কাঁদা বা ময়লা লেগে আছে। হাওর অঞ্চলে এরকম অবস্থায় বাবা মা রা কীভাবে শিশুদের পরিচ্ছন্ন রাখে? শিশুকে পরিষ্কার পরিচ্ছন্ন রাখতে অভিভাবকরা কী ধরণের সমস্যার সম্মুখীন হয়? এ ধরণের সমস্যাগুলো কীভাবে অতিক্রম করা সম্ভব এবং কে তাদেরকে এ ব্যাপারে সাহায্য করতে পারে? (Probe: পরিবার, সমাজ, সরকার, অন্যান্য প্রতিষ্ঠান যেমন ব্র্যাক)

6. Being able to be mobile in different seasons

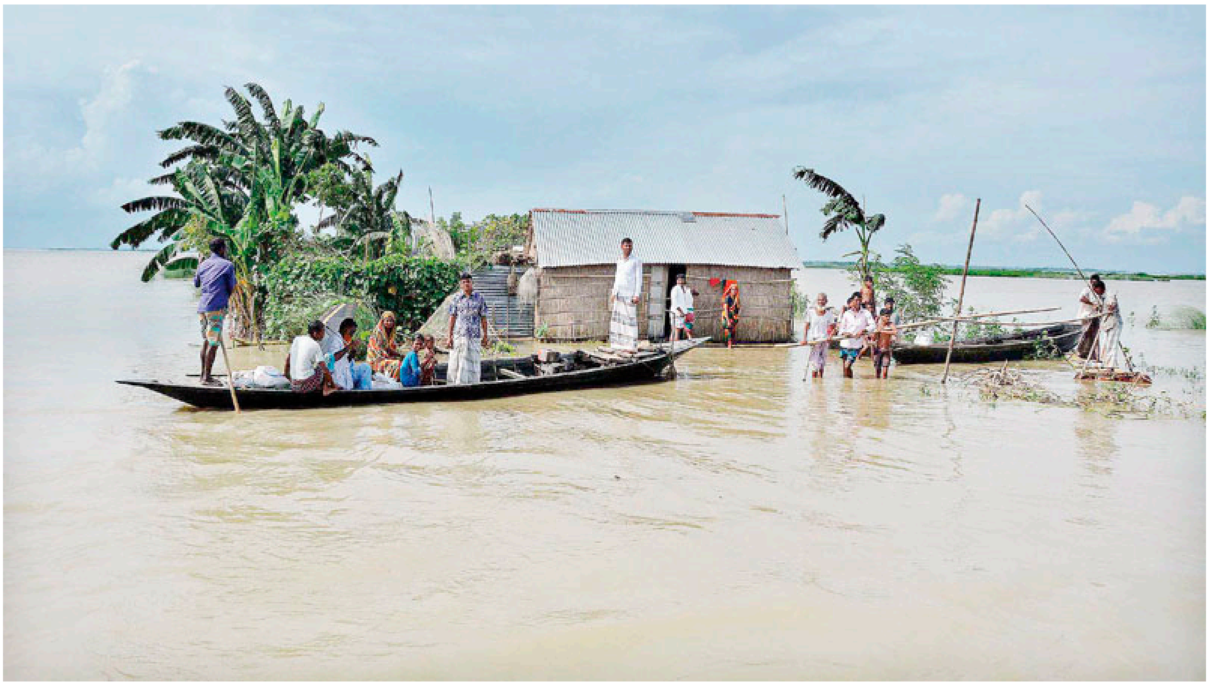

Pic 29: Mobility during flood (sourced from: http://m.theindependentbd.com/post/109022)

Question: In the picture, we see how people are struggling to move from one place to others when the areas are affected by floods. In such situation what kind of challenges do people face in haor? What happens to the mother and the children if their mobility is affected? Similarly during dry season when the roads are broken what do people face? What do they endure during the transitions from dry to wet or wet to dry season? How these challenges could be addressed? (Probe: family, community, govt., other institutions including BRAC)

প্রশ্নঃ এই ছবিতে আমরা দেখতে পাচ্ছি, যখন এলাকাগুলো বন্যা প্লাবিত থাকে তখন কীভাবে মানুষের এক জায়গা ঠেকে আরেক জায়গায় যাতায়াত করতে কষ্ট হয়। এরকম অবস্থায় মানুষ কী কী ধরণের সমস্যার সম্মুখীন হয়ে থাকে? যাতায়াতে সমস্যার সম্মুখীন হলে মা এবং শিশুদের কী অবস্থা হয়? একই ভাবে শুষ্ক মৌসুমে যখন রাস্তাঘাট ভাঙ্গা থাকে তখন মানুষ কী কী সমস্যার সম্মুখীন হয়? আর শুকনো মৌসুম থেকে বন্যার মাঝের সময় বা বন্যার পরে শুকনো মৌসুম আসার মাঝের সময় টুকো কী কী সমস্যা হয়ে থাকে? এ সমস্যা গুলো কীভাবে মোকাবেলা করা সম্ভব? (Probe: পরিবার, সমাজ, সরকার, অন্যান্য প্রতিষ্ঠান যেমন ব্র্যাক)

7. Being able to secure a source of safe drinking water for the family

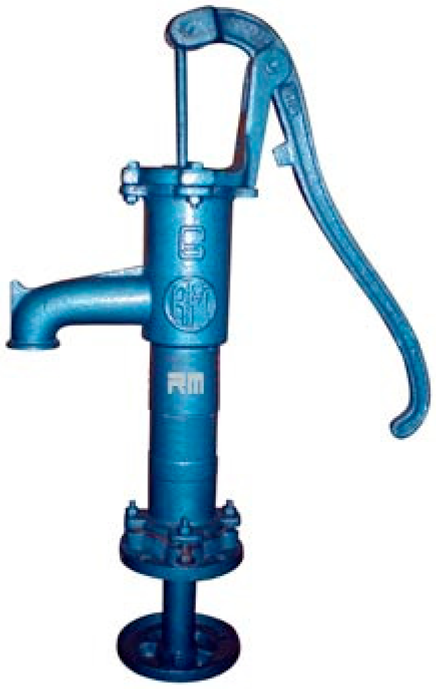

Pic 30: Tube well (sourced from: https://www.sstsbd.org/tube-well/)

Question: In the above picture we see a tube well from where people source safe drinking water. In haor areas how do people source safe drinking water for their children and family? What kind of challenges do the people face in haor to access safe drinking water and why? How the challenges could be addressed, who can help the haor dwellers in addressing the challenges and how? (Probe: family, community, govt., other institutions including BRAC)

প্রশ্নঃ এই ছবিতে আমরা একটি টিউবওয়েল দেখতে পাচ্ছি যেটা নিরাপদ খাবার পানির একটি উৎস। হাওর এলাকায় মানুষ কীভাবে তার পরিবার এবং শিশুদের জন্য নিরাপদ খাবার পানির ব্যাবস্থা করে? এসব এলাকায় নিরাপদ খাবার পানির জন্য মানুষ কী কী ধরনের সমস্যার সম্মুখীন হয়ে থাকে? এ ধরনের সমস্যাগুলো কীভাবে মোকাবেলা করা সম্ভব? কারা এ ব্যাপারে হাওর এলাকার মানুষদের সাহায্য করতে পারে এবং কীভাবে? (Probe করুনঃ পরিবার, সমাজ, সরকার, অন্যান্য প্রতিষ্ঠান যেমন ব্র্যাক)

We already came to the end of the interview, before we conclude the discussion, would like to add on anything that remain undiscussed? Thanks for your valuable inputs and participation.

আমরা সাক্ষাৎকারের একেবারে শেষ পর্যায়ে চলে এসেছি। শেষ করার আগে আপনি কি আরও কিছু বলতে চান যেটা আজকে আলোচনা হয় নি? সাক্ষাৎকারে অংশগ্রহন করে আপনার মূল্যবান মতামত প্রদানের জন্য ধন্যবাদ জানাই

© 2020 by the authors. Licensee MDPI, Basel, Switzerland. This article is an open access article distributed under the terms and conditions of the Creative Commons Attribution (CC BY) license (http://creativecommons.org/licenses/by/4.0/).
